# Interaktionsbezogene Stressoren und Ressourcen – Entwicklung einer Taxonomie zur menschengerechten Gestaltung von Interaktionsarbeit

**DOI:** 10.1007/s41449-023-00364-8

**Published:** 2023-05-15

**Authors:** Jonas Wehrmann

**Affiliations:** Fachbereich 1. Arbeitswelt im Wandel, Bundesanstalt für Arbeitsschutz und Arbeitsmedizin, Friedrich-Henkel-Weg 1–25, 44149 Dortmund, Deutschland

**Keywords:** Interaktionsarbeit, Dienstleistungsarbeit, Stressoren und Ressourcen, Psychische Belastung, Taxonomie, Interactive work, Service work, Stressors and resources, Mental stress, Taxonomy

## Abstract

Auch wenn sich bereits verschiedene Studien mit den besonderen Anforderungen von Interaktionsarbeit auseinandersetzen, wurden diese kaum aus einer integrierten Stressoren- und Ressourcenperspektive betrachtet (Bednarek 2014). So konzentriert sich die bisherige Forschung vor allem darauf, KundInnen in ihrer Rolle als Stressor zu untersuchen.

Ziel des Beitrags ist es, interaktionsbezogene Stressoren und Ressourcen zu identifizieren, zu systematisieren und diese hinsichtlich ihrer Relevanz für eine menschengerechte Arbeitsgestaltung von Interaktionsarbeit zu reflektieren. So wird das Forschungsfeld zunächst mittels einer systematischen Literaturanalyse erschlossen und anschließend eine explorativ‐qualitative Studie durchgeführt. Die Ergebnisse zeigen, dass interaktionsbezogene Stressoren vor allem aus unhöflichem oder aggressivem Kundenverhalten, hohen Kundenansprüchen sowie traumatischen Erfahrungen resultieren. Interaktionsbezogene Ressourcen beziehen sich auf die Interaktion mit freundlichen KundInnen, die Dienstleistende bei ihrer Arbeit unterstützen und dazu beitragen, dass diese ihre Tätigkeit als sinnstiftend erleben. Konkrete Gestaltungsfaktoren schließen u. a. eine ausreichende Zeit- und Personalbemessung sowie interaktionsdienliche Arbeitsmittel ein.

*Praktische Relevanz*: Die Studie schafft mit der Entwicklung einer branchen- und berufsübergreifenden Taxonomie einen konzeptionellen Rahmen, um Interaktionsarbeit gezielter menschengerecht gestalten zu können. Hierbei werden vier Themenfelder mit konkreten Gestaltungsfaktoren für interaktive Tätigkeiten aufgezeigt.

## Abstract (en)

Although various studies deal with the special demands of interactive work, these have only rarely been examined from an integrated stressor and resource perspective (Bednarek 2014). Thus, previous research concentrated on investigating customers in their role as stressors.

The aim of this article is to identify and categorise interaction-related stressors and resources and reflect their role in order to design interactive work in a humane and health-promoting way. As a first step, the research field was explored by means of a systematic literature analysis. Based on the results an explorative-qualitative study was conducted. The results show that interaction-related stressors particularly result from unfriendly or aggressive customer behaviour, high customer demands and traumatic experiences. Interaction-related resources refer to friendly clients who support service providers in their work and help them to experience their work as meaningful. Work design aspects include sufficient time and human resources as well as interaction-supporting work equipment.

*Practical relevance*: With the development of a comprehensive taxonomy independent of economic sectors or occupations, the study creates a conceptual framework to design interactive work in a humane and health-promoting way. Four thematic fields with concrete design factors for interactive work are identified.

## Ausgangslage

Eine Vielzahl von Dienstleistenden verbringt einen Großteil ihrer Arbeit in der Interaktion mit anderen Menschen (Dormann und Zapf 2004; Holz et al. 2004). Die zu leistende Interaktionsarbeit mit KundInnen, PatientInnen und KlientInnen bildet dabei einen wichtigen Bestandteil ihrer täglichen Arbeit (Dormann und Zapf 2004).^1^ Wenngleich viele Beschäftigte ihre Tätigkeit als abwechslungsreich empfinden und den zwischenmenschlichen Austausch schätzen, kann die Interaktion mit KundInnen, PatientInnen und KlientInnen für Dienstleistende mit Konflikten verbunden sein (Dormann und Zapf 2004; Holz et al. 2004). Nicht selten sind KundInnen, PatientInnen und KlientInnen unhöflich, haben überzogene Erwartungen oder sind sogar aggressiv und sehen sich dabei im Recht (Grandey et al. 2004; Harris und Reynolds 2004; Walsh 2011). Dabei stellen diese konfliktreichen Erlebnisse mit KundInnen, PatientInnen und KlientInnen keineswegs Einzelfälle dar, sondern gehören zum Arbeitsalltag von Dienstleistenden. So konnten Bitner et al. (1994) in ihrer Studie zeigen, dass 22 % der von Beschäftigten genannten negativen Events im Zusammenhang mit problematischen KundInnen standen. In Anbetracht der Tatsache, dass Dienstleistende in der Regel wesentlich mehr Zeit mit KundInnen, PatientInnen und KlientInnen verbringen als mit ihren KollegInnen oder Vorgesetzten, gehen Dormann und Zapf (2004) davon aus, dass Konflikten mit KundInnen, PatientInnen und KlientInnen eine genauso große, wenn nicht sogar größere Bedeutung zukommt. Trotz der Relevanz des Themas kommen Glaser et al. (2008, S. 15) zu dem Schluss, dass „spezifische Anforderungen und Belastungen der Interaktionsarbeit […] bislang nur wenig im Blickfeld der arbeitswissenschaftlichen Forschung [standen]“.

Neben den negativen Seiten der Interaktionsarbeit bietet die Arbeit mit Menschen ebenfalls vielfältige Ressourcen. Viele Beschäftigte in Dienstleistungsberufen wählen ihren Beruf bewusst aus, weil sie mit Menschen arbeiten wollen (Grandey et al. 2005; Zimmermann et al. 2011). Für sie stellt die Arbeit mit KundInnen, PatientInnen und KlientInnen damit eine zentrale Motivation ihrer Berufswahl dar (Homburg und Stock 2004; Zimmermann et al. 2011). Viele Dienstleistende haben Freude an der Interaktion mit KundInnen, KlientInnen und PatientInnen, erleben Ihre Tätigkeit als sinnstiftend und schätzen insofern vor allem den sozialen Anteil ihrer Arbeit (Holz et al. 2004). Während in der Vergangenheit bestehende Studien zum Ziel hatten, die negativen Seiten der Arbeit mit Menschen zu untersuchen, wurde die Interaktion zwischen Dienstleistenden und KundInnen, PatientInnen und KlientInnen als mögliche Quelle von Wohlbefinden und psychischer Gesundheit bislang nur vereinzelt untersucht (Loera et al. 2016; Zimmermann et al. 2011). So wurden KundInnen in der bisherigen Forschung vor allem in ihrer Rolle als potenzieller Stressor betrachtet. In diesem Kontext wurde u. a. untersucht, wie sich aggressives (Grandey et al. 2004), unfreundliches (Walsh 2011; Yagil 2021) oder unfaires Kundenverhalten (Berry und Seiders 2008) auf die Gesundheit und das Befinden von Beschäftigten auswirkt. Weniger betrachtet wurde hierbei jedoch, inwieweit KundInnen mit ihrem positiven Verhalten eine Ressource in der Dienstleistungsinteraktion darstellen können (Loera et al. 2016).

In der Zusammenschau weisen die bisherigen Erkenntnisse darauf hin, dass zumeist aus einer pathogenetischen Perspektive erforscht worden ist, welche gesundheitsbeeinträchtigenden Faktoren mit der zu leistenden Interaktionsarbeit verbunden sind. Aus einer salutogenetischen Betrachtung wurden bisher jedoch kaum Erkenntnisse zu den positiven und gesundheitsförderlichen Merkmalen der Arbeit an und mit Menschen untersucht (Bednarek 2014; Nerdinger 2019; Zimmermann et al. 2011). Vor diesem Hintergrund erscheint es notwendig, eine ganzheitliche Perspektive auf den Forschungsgegenstand der Interaktionsarbeit zu richten und neben Stressoren ebenfalls Ressourcen mit in die analytische Betrachtung einzubeziehen.

Ziel des Beitrags ist es, interaktionsbezogene Stressoren und Ressourcen zu identifizieren, zu systematisieren und diese hinsichtlich ihrer Relevanz für eine menschengerechte Arbeitsgestaltung von Interaktionsarbeit zu reflektieren. Damit leistet der vorliegende Artikel nicht nur einen Beitrag, interaktionsbezogene Stressoren und Ressourcen tiefergreifend zu erforschen, sondern schafft ebenfalls einen konzeptionellen Rahmen, um Interaktionsarbeit im Sinne einer prospektiven Arbeitsgestaltung menschengerecht gestalten zu können. Mit der Entwicklung einer Taxonomie gestaltungsrelevanter Faktoren für Interaktionsarbeit kommt der Artikel der arbeitswissenschaftlichen Forderung nach einer „branchen- und berufsübergreifende[n] Klassifikation von Merkmalen der Arbeit […], die für die menschengerechte Gestaltung interaktiver Tätigkeiten relevant sind,“ nach (Tisch et al. 2020, S. 44). Nicht zuletzt soll ein theoretischer Beitrag geleistet werden, die modelltheoretischen Annahmen des JD‑R Modells durch eine Multi-Level-Perspektive zu erweitern (Demerouti et al. 2001).

Im vorliegenden Artikel werden zunächst theoretische Grundlagen beschrieben, bevor das methodische Vorgehen dargelegt und anschließend zentrale Ergebnisse der Literaturanalyse sowie der qualitativen Studie vorgestellt und diskutiert werden. Der Fokus des Beitrags wird auf die Ergebnisse der qualitativen Studie gelegt.

## Das Job-Demands-Resources-Modell als theoretische Grundlage

Die Wahl des Job-Demands-Resources-Modells (JD‑R Modell) als übergeordnetes konzeptionelles Rahmenmodell ist darin begründet, interaktionsbezogene Ressourcen weiter in den Vordergrund der wissenschaftlichen Betrachtung zu stellen. Die Passung des JD‑R Modells für diese Studie drückt sich vor allem in dessen flexibler Anwendung aus (Demerouti et al. 2001). So können sämtliche Arbeitsbedingungen und Tätigkeitsprofile unterschiedlicher Berufsgruppen anhand von zwei weitgehend unabhängigen Dimensionen (Job Demands und Job Resources) abgebildet werden (Stock-Homburg et al. 2010). Neben der Identifizierung relevanter Stressoren und Ressourcen stellt das Modell mit seinen implizierten Wirkungsannahmen einen nützlichen theoretischen Rahmen dar, um die Folgen von interaktionsbezogenen Stressoren und Ressourcen für die Gesundheit und das Wohlbefinden von Beschäftigten zu betrachten. Darüber hinaus legt das JD‑R Modell mit seinen implizierten Annahmen einen definitorischen Rahmen für die begriffliche Eingrenzung interaktionsbezogener Stressoren und Ressourcen fest:

- *Interaktionsbezogene Stressoren: „Faktoren, die aus der Interaktionsarbeit resultieren und mit physischen oder psychischen Kosten für die Beschäftigten verbunden sind.“*
- *Interaktionsbezogene Ressourcen:* „*Faktoren, die aus der Interaktionsarbeit resultieren und mit positiven Folgen für das Befinden und die Gesundheit von Beschäftigten einhergehen und funktional für das Erreichen von arbeitsbezogenen Zielen sind und negative Wirkungseffekte von Stressoren reduzieren können.“*

**Figure 1:**
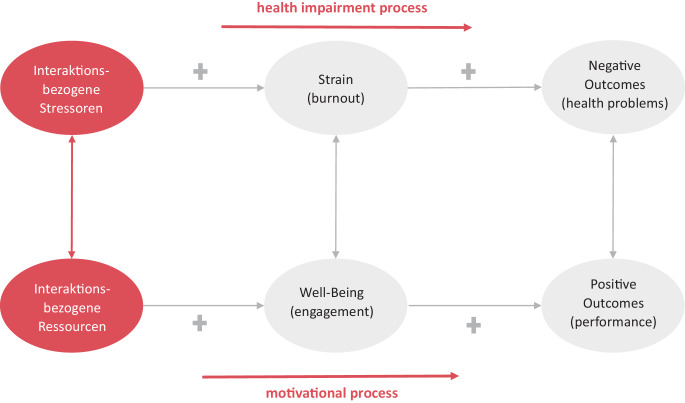

**Figure 2:**
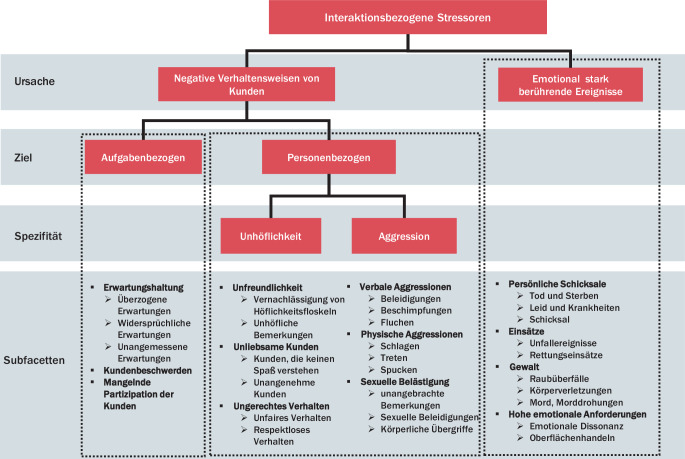

In Anbetracht der weitgehenden Vernachlässigung von Ressourcen in der Forschung zu Interaktionsarbeit wird in der vorliegenden Arbeit auf die konzeptionelle Weiterentwicklung des JD‑R Modells von Schaufeli und Taris (2014) zurückgegriffen (Abb. 1). Mit ihrem überarbeiteten Modell erklären die Autoren nicht nur aus einer pathogenetischen Perspektive langfristige negative gesundheitliche Folgen, sondern geben ebenfalls Hinweise dazu, wie sich Arbeit in ihren langfristigen Folgen positiv auf die Gesundheit und das Wohlbefinden von Beschäftigten auswirken kann. Die Autoren unterscheiden hinsichtlich des „health impairment- und motivational processes“:

- *„health impairment process“:* Schaufeli und Taris (2014) gehen in ihrem überarbeiteten Modell davon aus, dass die Kombination hoher arbeitsbezogener Stressoren und geringer arbeitsbezogener Ressourcen mit verschiedenen negativen, kurz- bis mittelfristigen Wirkungen (z. B. Burnout) assoziiert sind, die je nach Art und Ausprägung zu langfristigen gesundheitlichen Folgen wie bspw. Depressionen, Herz-Kreislauf-Erkrankungen oder psychosomatischen Beschwerden führen können (Abb. 2)
- *„motivational process“:* Analog dazu gehen Schaufeli und Taris (2014) davon aus, dass ausreichend zur Verfügung stehende Ressourcen dazu beitragen, dass arbeitsbezogene Ziele erreicht werden und infolgedessen kurz- bis mittelfristig zu Veränderungen des Arbeitsengagements führen können. Die Autoren nehmen an, dass ein gesteigertes Engagement langfristig mit verschiedenen positiven Wirkungsfolgen wie einem hohen Leistungsempfinden oder einem gesteigerten Sinnerleben assoziiert ist (Abb. 2)

Im weiteren Verlauf dieser Arbeit soll das JD‑R Modells als Rahmenmodell für die Identifizierung relevanter interaktionsbezogener Stressoren und Ressourcen sowie möglicher Wirkungsfolgen dienen. Das Modell lässt zunächst offen, welche Stressoren und Ressourcen aus der Interaktion mit KundInnen, PatientInnen und KlientInnen resultieren. Ziel der Literaturanalyse und der qualitativen Studie ist es, das vorgestellte Modell empirisch mit „Leben zu füllen“.

## Forschungsdesign und Methodologie

Da sich der gegenwärtige Forschungsstand interaktionsbezogener Stressoren und Ressourcen bis dato fragmentiert darstellt und eine Systematisierung bestehender Erkenntnisse bislang nicht stattgefunden hat, wurde ein exploratives Mixed-Methods-Design gewählt.^2^ So wurde das Forschungsfeld zunächst mittels einer systematischen Literaturrecherche erschlossen, um den aktuellen Forschungsstand interaktionsbezogener Stressoren und Ressourcen darstellen zu können. Anschließend wurde eine explorativ-qualitative Studie (halbstandardisierte Interviews & teilnehmende Beobachtungen) über verschiedene Berufsgruppen durchgeführt, um im Zuge eines induktiven Vorgehens zentrale interaktionsbezogene Stressoren und Ressourcen zu identifizieren. Eine methodische Verknüpfung beider Forschungsansätze konnte dadurch realisiert werden, dass die Ergebnisse der Literaturanalyse einen unmittelbaren Einfluss auf die Entwicklung des Interview- und Beobachtungsleitfadens hatten. Dieser multimethodische Feldzugang eröffnete die Gelegenheit, den Forschungsgegenstand aus verschiedenen Perspektiven methodisch und methodologisch fundierter zu betrachten (Creswell 1999; Mayring 2015).

### Methodisches Vorgehen bei der Literaturanalyse

Die Aufbereitung des Forschungsstandes folgte dem Vorgehen einer systematischen Literaturanalyse. So wurden zunächst geeignete Literaturquellen ausgewählt und relevante Suchbegriffe formuliert, bevor die eigentliche Literaturrecherche und anschließend die Darstellung der Ergebnisse erfolgten. Für die Recherche wurden die psychologischen Datenbanken PsychARTICLES, PsychINFO und PSYNDEX unter Zuhilfenahme der Rechercheplattform Ebscohost ausgewählt und ergänzend dazu die Online-Suchmaschine Google Scholar hinzugezogen. Darüber hinaus wurden im Rahmen einer Schneeballsuche identifizierte Studien auf relevante Literaturquellen hin überprüft.^3^

Bei der Eingrenzung der Suchbegriffe zeigte eine erste Sichtung, dass Interaktionsarbeit häufig nicht explizit, sondern vielmehr implizit in unterschiedlichen Forschungsdebatten unter verschiedenen Begrifflichkeiten diskutiert wird (s. Tisch et al. 2020; Dörflinger 2022). Dadurch war es schwierig, feste Suchbegriffe für ein rein systematisches Vorgehen zu definieren. Deshalb wurden neben der Suche nach „interaktionsbezogenen Stressoren und Ressourcen“ – als feststehender Suchbegriff – ebenfalls Synonyme formuliert (Tab. 2 im Anhang). Eine erste Grundlage für die Bestimmung von Suchbegriffen bildete die Literaturrecherche von Bednarek (2014) zu positivem und negativem Kundenverhalten sowie ein einführender Überblicksartikel von Holz et al. (2004) zu sozialen Stressoren in der Arbeitswelt. Damit handelt es sich bei der vorliegenden Literaturanalyse nicht um eine abschließende Darstellung des Forschungsstandes, sondern es soll vielmehr ein erster Überblick zu interaktionsbezogenen Stressoren und Ressourcen gegeben werden, um Themeninhalte für den halbstandardisierten Leitfaden der aufbauenden Beobachtungs- und Interviewstudie zu identifizieren. Studien wurden ausgewählt, sofern sie folgende Einschlusskriterien erfüllten:

1. Veröffentlichung in einschlägigen wissenschaftlichen Zeitschriften (Peer-Review-Verfahren).
2. Zeitraum der Veröffentlichung: 2002 bis 2022, da in den letzten zwei Dekaden Interaktionsarbeit als Forschungsgegenstand in der arbeitswissenschaftlichen Debatte zunehmend Aufmerksamkeit geschenkt wurde.
3. Studien mit Fokus auf Führungskräfte oder KollegInnen wurden nur dann berücksichtigt, wenn diese für die Entstehung und Wirkung interaktionsbezogener Stressoren und Ressourcen im Kontext der Interaktion mit Externen (KundInnen, PatientInnen) relevant waren.
4. Studienkontext: Erwerbstätigkeit (kein privater Kontext).
5. In die Analyse wurden sowohl qualitative als auch quantitative Studien und konzeptionelle Veröffentlichungen eingeschlossen.

Im Rechercheprozess wurden die Studien zunächst anhand des Titels und des Abstracts gesichtet. Erwiesen sich die Studien als relevant, wurden diese hinsichtlich verschiedener Merkmale (u. a. Studiendesign, Stichprobengröße, betrachtete Konstrukte, untersuchte Berufsgruppen sowie zentrale Studienergebnisse) systematisiert.

### Methodisches Vorgehen bei der qualitativen Studie

Ziel der qualitativen Studie war es, im Zuge eines induktiv geleiteten Vorgehens ein tiefgreifendes, subjektbezogenes Verständnis von interaktionsbezogenen Stressoren und Ressourcen zu erhalten und auf Basis der Erfahrungen und Einschätzungen der Beschäftigten Rückschlüsse auf gestaltungsrelevante Faktoren für Interaktionsarbeit zu ziehen. Darüber hinaus diente die qualitative Studie dazu, ein besseres Verständnis von den arbeitsorganisatorischen Rahmenbedingungen, unter denen Interaktionsarbeit erbracht wird, zu erhalten.

Im Rahmen der halbstrukturierten Experteninterviews wurden im Sinne der Berücksichtigung möglichst diverser Perspektiven neben Beschäftigten ebenfalls VertreterInnen des Managements, des Betriebs- und Personalrats sowie der Arbeitgeber- und Arbeitnehmerverbände befragt. Dabei wurde angesichts der Tatsache, dass Interaktionsarbeit in verschiedenen beruflichen Kontexten und Tätigkeitsbereichen geleistet wird, das Format einer vergleichenden Studie gewählt (Tisch et al. 2020; Böhle und Glaser 2006). Dieses methodische Vorgehen erschien insbesondere vor dem Hintergrund der Entwicklung einer branchen- und berufsübergreifenden Taxonomie von Faktoren für die menschengerechte Gestaltung von Interaktionsarbeit erforderlich, da in der bisherigen Forschung vor allem einzelne Berufsgruppen betrachtet wurden.

Um eine transparente und zielgerichtete Auswahl der Beschäftigtengruppen zu ermöglichen, wurden vorab Auswahlkriterien identifiziert, die dabei helfen sollten, die Vielzahl an Beschäftigten, die Interaktionsarbeit leisten, sinnvoll zu unterscheiden.^4^ Hierbei wurden vor allem Beschäftigtengruppen ausgewählt, die sich durch einen hohen interaktiven Tätigkeitsanteil auszeichneten. Die Auswahlkriterien bezogen sich einerseits auf Beschäftigtengruppen, die mit verschiedenen betriebsexternen Personengruppen zusammenarbeiteten (u. a. KundInnen, PatientInnen, KlientInnen, BürgerInnen und Gäste) und andererseits sollten verschiedene Charakteristika der zu leistenden Interaktionsarbeit (u. a. Dauer, Frequenz sowie Inhalt der Interaktion) berücksichtigt werden. Auf Grundlage der abgeleiteten Kriterien wurden insgesamt sechs Beschäftigtengruppen ausgewählt: Pflegekräfte, Beschäftigte im Einzelhandel, Beschäftigte in der Gastronomie, UnternehmensberaterInnen, PolizistInnen sowie FallmanagerInnen der Bundesagentur für Arbeit.

Ergänzend zu den Interviews wurden neun teilnehmende Beobachtungen in den befragten Organisationen von zwei Forschenden des Projektteams durchgeführt, wobei diese in der Regel zwischen zwei Interviewblöcken stattfanden (Tab. 1). Damit ermöglichte das methodische Vorgehen es einerseits, Inhalte vorheriger Interviews validierend zu überprüfen und andererseits, tiefergreifende Erkenntnisse gewinnen zu können, die durch die alleinige methodische Anwendung von Experteninterviews nicht zugänglich gewesen wären (z. B. Einsicht in die Arbeitsumgebung). Dabei wurde in Vorbereitung auf die Beobachtungen ein Beobachtungsleitfaden entwickelt (Tab. 3 im Anhang). Die Beobachtungen wurden protokolliert und zusammen mit den Interviewtranskripten softwarebasiert mit Hilfe des Programms MAXQDA 2020 ausgewertet. Beobachtungen und Interviews ergänzten sich dabei mit dem Ziel, interaktionsbezogene Stressoren und Ressourcen differenzierter zu betrachten und psychologisch bewerten zu können (Glaser et al. 2008).

**Table 1:** 

Beschäftigtengruppe	Differenzierungsmerkmale	Anzahl der teilnehmenden Organisationen	Anzahl der Interviews (physisch/virtuell)	Anzahl der Beobachtungen	Erhebung der Daten
Beschäftigte in der Altenpflege	Stationäre und ambulante Altenpflege	3	14 (12/2)	2	ForscherIn 1
Servicekräfte in der Gastronomie	Einzel‑, System und Betriebsgastronomie	4	23 (21/2)	4	
Beschäftigte im Einzelhandel	Baumärkte, Möbelhäuser	2	14 (10/4)	2	
UnternehmensberaterInnen	Selbstständige und abhängig beschäftigte BeraterInnen	19	29 (0/29)	1	ForscherIn 2/ForscherIn 1
FallmanagerInnen bei der BA	Jobcenter in Städten mit unterschiedlichen Problemlagen	3	18 (0/18)	0^a^	ForscherIn 3/ForscherIn 1
PolizistInnen	Polizeikommissariate im ländlichen und kleinstädtischen Raum	2	8 (4/4)	0^a^	
**Gesamt**		**33**	**106**	**9**	

^a^Aufgrund der Sars-Cov-2-Pandemie waren Beobachtungen bei der Polizei und dem Fallmanagement nicht möglich

Insgesamt wurden 106 Personen in 6 Beschäftigtengruppen und 33 Organisationen in Deutschland befragt (Tab. 1). Die Datensammlung erstreckte sich dabei von 2020 bis 2022. Die durchschnittliche Dauer der Interviews umfasste 1,5 h. Die Interviews wurden von drei Forschenden des Forschungsteams erhoben und anschließend durch einen Transkriptionsdienstleister verschriftlicht und handschriftlich protokolliert. Aufgrund der Sars-Cov‑2 Pandemie und des erschwerten Feldzugangs wurden die Interviews sowohl online als auch vor Ort in den Organisationen durchgeführt (Tab. 1). Um die Interviews in Präsenz durchführen zu können, wurde ein umfassendes Hygienekonzept erarbeitet.

Im Zuge der Auswertung wurde auf die qualitative Inhaltsanalyse nach Mayring (2015) zurückgegriffen. Auf Basis der induktiven Kategorienbildung wurde das Interviewmaterial zunächst in seine Bestandteile zerlegt und anschließend mit einem am Datenmaterial entwickelten Kodierungssystem bearbeitet. Das induktiv aus dem Material entwickelte Kategoriensystem gab dabei vor, welche Inhalte aus dem Interviewmaterial extrahiert werden sollten. Durch die Formulierung von klaren Auswertungsregeln wurde ein systematisches, nachvollziehbares und intersubjektiv überprüfbares methodisches Vorgehen sichergestellt (Mayring 2015).

## Ergebnisse der Literaturanalyse

Als übergeordnetes Ergebnis der Literaturrecherche lässt sich festhalten, dass eine übergreifende Einordung und Systematisierung interaktionsbezogener Stressoren und Ressourcen in der Forschung bislang nur bedingt stattgefunden hat und die verwendeten Begrifflichkeiten zur Beschreibung dieser Stressoren recht heterogen waren. Im Folgenden werden ausgewählte Ergebnisse der Literaturanalyse zu interaktionsbezogenen Stressoren und Ressourcen vorgestellt. In Rückbezug zu dem vorgestellten JD‑R Modell sollen dabei auch mögliche Wirkungsfolgen dargestellt werden.

### Ergebnisse im Hinblick auf interaktionsbezogene Stressoren

Auf Grundlage der Literaturanalyse wurden 28 Studien identifiziert, die sich mit interaktionsbezogenen Stressoren auseinandersetzten (Tab. 4 im Anhang). Die Studienlage war durch Heterogenität und Multidimensionalität geprägt. Es konnten zwei übergeordnete Themenfelder interaktionsbezogener Stressoren identifiziert werden, die sich wiederum hinsichtlich weiterer Subfacetten untergliedern ließen: 1) negative Verhaltensweisen, 2) emotional stark berührende Ereignisse.

#### Ergebnisse zur Wirkung interaktionsbezogener Stressoren basierend auf den Annahmen des JD-R Modells

Die Ergebnisse der Literaturanalyse zeigen, dass Studien bisher vor allem den Zusammenhang zwischen interaktionsbezogenen Stressoren und den drei Subfacetten des Burnout-Syndroms untersuchten (8 von 28 Studien). So konnten Grandey et al. (2004) zeigen, dass die Häufigkeit der verbalen Aggression von KundInnen im Zusammenhang mit der emotionalen Erschöpfung der Dienstleistenden stand. Sliter et al. (2010) weisen in ihrer Studie nach, dass unhöfliches Kundenverhalten positiv mit der emotionalen Erschöpfung und negativ mit der subjektiv wahrgenommenen Arbeitsleistung zusammenhängt. Dormann und Zapf (2004) zeigen, dass kundenbezogene soziale Stressoren positiv mit den Burnout-Subfacetten und psychosomatischen Beschwerden sowie negativ mit der Arbeitszufriedenheit von Dienstleistenden in Zusammenhang stehen. Unhöfliche oder auch respektlose KundInnen haben nicht nur einen Einfluss auf das Befinden und die Gesundheit von Beschäftigten, sondern können ebenfalls deren Leistungsfähigkeit beeinflussen. So berichten Sliter et al. (2012) im Zuge ihrer an Bankangestellten durchgeführten Befragung, dass die Interaktion mit unhöflichen KundInnen sowohl eine geringere Verkaufsleistung als auch eine Zunahme der Ausfalltage der Beschäftigten zur Folge hatte. Die AutorInnen führen die geringere Verkaufsleistung darauf zurück, dass das unhöfliche Verhalten der KundInnen als Auslöser für ein abnehmendes Engagement und eine mangelnde Leistungsbereitschaft der Dienstleistenden gesehen werden kann. Weitergehend zeigt Walsh (2011), dass unhöfliches Kundenverhalten im Zusammenhang mit einer gesteigerten Kündigungsabsicht steht.

### Ergebnisse im Hinblick auf interaktionsbezogene Ressourcen

**Figure 3:**
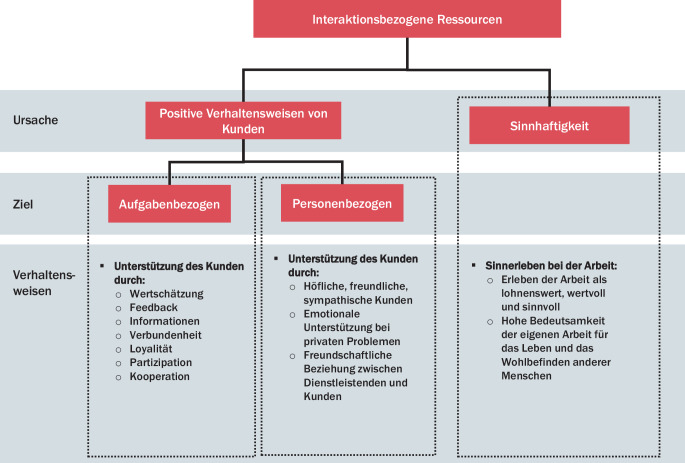

Auf Basis der Literaturanalyse konnten 9 Studien hinsichtlich interaktionsbezogener Ressourcen identifiziert werden (Tab. 5 im Anhang). Die Mehrheit dieser Arbeiten konzentrierte sich auf die spezifische Betrachtung einzelner Facetten interaktionsbezogener Ressourcen, wobei größtenteils Beschäftigte aus einzelnen Berufsgruppen und Branchen untersucht wurden. Die gewonnenen Erkenntnisse ließen sich zwei Themenfeldern zuordnen: 1) positive Verhaltensweisen von KundInnen, 2) Sinnhaftigkeit (Abb. 3).

#### Ergebnisse zur Wirkung interaktionsbezogener Ressourcen basierend auf den Annahmen des JD-R Modells

Im Rahmen der Literaturanalyse konnten nur vereinzelt Studien identifiziert werden, die die positive Wirkung von Interaktionsarbeit auf das Wohlbefinden und die Gesundheit von Beschäftigten betrachtet haben. So verweisen Nerdinger et al. (2019, S. 659) darauf, dass in der Dienstleistungsforschung „die positiven Wirkungen deutlich weniger Forschung hervorrufen“ – dies verdeutlicht nicht zuletzt das Verhältnis von 9 zu 28 Studien. Zimmermann et al. (2011) zeigen, dass das unterstützende Verhalten von KundInnen mit der positiven Stimmung von Dienstleistenden assoziiert ist. Wegge et al. (2012) weisen in einem experimentellen Design im Bereich des Call-Centers nach, dass Beschäftigte, die mit höflichen KundInnen interagierten, mehr positive Emotionen und weniger emotionale Dissonanz im Vergleich zu Beschäftigten aufwiesen, die mit unfreundlichen KundInnen in Interaktion traten. Weiter konnten Kim und Yoon (2012) in ihrer im Einzelhandel durchgeführten Studie bestätigen, dass die positiven Emotionen von KundInnen zu einem positiven Affekt der Dienstleistenden führte. Yi et al. (2011) berichten, dass unterstützende Kundenverhaltensweisen in einem positiven Zusammenhang mit der Arbeitszufriedenheit, der Arbeitsleistung sowie dem Commitment und negativ mit der Kündigungsabsicht von Dienstleistenden stehen.

## Zentrale Ergebnisse der qualitativen Studie (Experteninterviews und teilnehmende Beobachtung)

**Figure 4:**
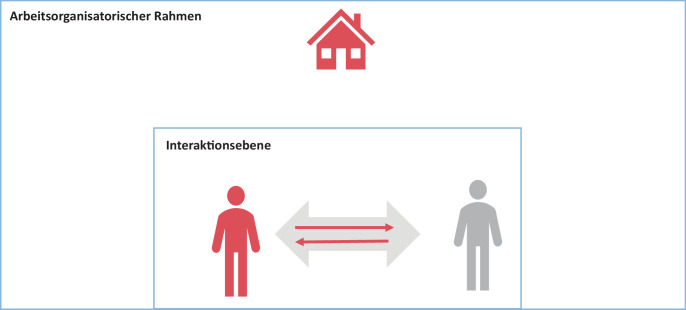

Ziel war es, eine branchen- und berufsübergreifende Taxonomie von Faktoren für die menschengerechte Gestaltung von Interaktionsarbeit zu entwickeln. Die hierarchische Struktur des Kategoriensystems, abgeleitet aus den Interviews und den teilnehmenden Beobachtungen, ermöglichte es nicht nur, Stressoren und Ressourcen auf der Interaktionsebene, sondern ebenfalls gestaltungsrelevante Faktoren auf der arbeitsorganisatorischen Ebene zu betrachten (Abb. 4).

### Stressoren auf der Interaktionsebene

Auf der Interaktionsebene ließen sich fünf Hauptkategorien von Stressoren identifizieren, die sich aus der direkten Interaktion zwischen Dienstleistenden und KundInnen, PatientInnen oder KlientInnen ergaben (Tab. 6 im Anhang).

#### Negative personenbezogene Kundenverhaltensweisen

Auf Basis der Interviews konnten zwei übergeordnete Formen von negativen personenbezogenen Kundenverhaltensweisen identifiziert werden: unhöfliches und aggressives Verhalten. Unhöfliches Kundenverhalten zeigte sich darin, dass KundInnen die Dienstleistenden duzten, nicht begrüßten oder sich für den erbrachten Service nicht bedankten. Beschäftigte nahmen KundInnen insbesondere dann als unhöflich wahr, wenn diese sich *ungerecht *und* unfair* gegenüber den Dienstleistenden verhielten oder keinen Respekt zeigten: *„Die haben doch eh alle keine Ahnung. Sonst würden sie ja nicht im Baumarkt arbeiten. Die dummen Baumarktverkäufer“ *(*Beschäftigte eines Baumarkts*). Aggressive Kundenverhaltensweisen konnten hinsichtlich verbaler (Beschimpfen, Beleidigen, Fluchen, Gewaltandrohungen), sexueller (unangebrachte Bemerkungen, sexualisierte Witze, unangemessenen Berührungen) und physischer Aggression (Schlagen, Bespucken, Werfen mit Gegenständen) unterschieden werden. So schildert der Beschäftigte eines Fastfood-Restaurants: „*Die Situation war mit einem Kunden, der einen Latte Macchiato bestellt hatte, der dann viel zu kalt war. Der hat dann den Mitarbeiter rausgerufen und wollte sich dann mit dem schlagen. Und […] wir hatten hier drinnen mal jemanden, der das ganze Tablett einfach [auf mich] raufgeschmissen hat“.* Gemein ist den zuvor genannten Verhaltensweisen, dass sie in der Regel ein Verhalten beschreiben, welches sich durch die Verletzung allgemein akzeptierter sozialer Normen charakterisieren lässt.

#### Negative arbeitsbezogene Kundenverhaltensweisen

Während sich die zuvor beschriebenen negativen Verhaltensweisen gegen die Person des Dienstleistenden richtete, bezogen sich negative arbeitsbezogene Verhaltensweisen auf den Arbeitsgegenstand der erbrachten Dienstleistung. Im Rahmen der Interviews konnte eine große Bandbreite negativer arbeitsbezogener Verhaltensweisen identifiziert werden. So berichteten Beschäftigte vielfach von *überzogenen oder auch unangemessenen Erwartungen* von KundInnen (z. B. Einfordern von Sonderbehandlungen oder Zusatzleistungen). Zudem nahmen die Beschäftigten *illegitim erscheinende Beschwerden, eine mangelnde Partizipation, Abstimmungsprobleme mit KundInnen sowie Rachehandlungen *der KundInnen (z. B. beleidigende Kommentare im Internet) als Stressoren ihrer täglichen Arbeit wahr.

#### Traumatische Erfahrungen

umfassten extreme und außergewöhnlich stark emotional berührende Ereignisse im Arbeitsleben der Beschäftigten. Dazu zählte der Umgang mit Schicksal, Tod sowie schweren Krankheiten. Insbesondere im Bereich der Pflege waren Beschäftigte immer wieder mit dem Tod von PatientInnen konfrontiert: „*Da habe ich geheult […], da habe ich echt geheult, Rotz und Wasser. Und beim Zweiten denn auch. Vor allem, ich habe gesagt, der ist nicht tot, der LEBT. Weil der Arm fiel runter, die Luft kommt raus. Ich sagte: ‚Der lebt noch, das geht nicht‘“ (Beschäftigte stationäre Altenpflege).* Weitergehend umfassten traumatische Ereignisse die Konfrontation mit schweren Unfallereignissen sowie Rettungseinsätzen, Raubüberfällen oder die Herausforderung, schlechte Nachrichten überbringen zu müssen. Insbesondere Beschäftigte im Polizeidienst waren gefordert, bei tödlich verlaufenden Einsatzlagen Angehörigen Todesnachrichten überbringen zu müssen.

#### Stressoren aus der Emotionsarbeit

ergaben sich insbesondere aus der erlebten emotionalen Dissonanz. So berichteten die Beschäftigten, dass es zu ihrem Arbeitsalltag gehöre, eigene Gefühle unterdrücken zu müssen, um den Vorgaben der Organisation zu entsprechen. Insbesondere in Situationen, in denen KundInnen unfreundlich, herablassend oder auch beleidigend gegenüber den Beschäftigten auftraten, fiel es Dienstleistenden schwer, diesen weiterhin freundlich gegenüberzutreten. Dabei entwickelten die Beschäftigten ganz eigene Emotionsregulationsstrategien: *„Na ja, ich unterdrücke meine Gefühle so lange, bis ich gegen die Wand schlage. […] Wenn ich zum Beispiel einen sehr, sehr nervigen Kunden habe, der dann halt mir SO auf die Pelle rückt, dass ich halt kurz davor bin, meine Selbstbeherrschung zu verlieren, warte ich, bis er halt weg ist und dann schlage ich gegen meine Lieblingswand“ (Beschäftigter eines Fast Food Restaurants).*

#### Die Unplanbarkeit von Interaktionsarbeit

stellt ein grundlegendes Charakteristikum der Arbeit mit Menschen dar, welches häufig als unbeabsichtigte Anforderung im Arbeitsalltag von Beschäftigten auftritt und trotz vorrausschauender Planung nicht gänzlich ausgeschaltet werden kann. So wussten Beschäftigte nicht, wer die Dienstleistung in Anspruch nehmen wird: „*Das ist einfach auch ein Faktor, der, JA, Interaktionsarbeit belastet […] ich weiß nicht, WER kommt, das ist immer eine Überraschung […] Ich kann mich darauf nicht vorbereiten“ (Beschäftigter Betriebsgastronomie). *Weitergehend konnten Dienstleistende nicht antizipieren, wie sich KundInnen im nächsten Augenblick verhalten werden, sodass eine entsprechende Reaktion vorab nicht geplant werden konnte. Darüber hinaus traten immer wieder unvorhersehbare Ereignisse auf, die es für die Beschäftigten unmöglich machten, ihren Arbeitsalltag durchzuplanen. Zudem berichteten die Beschäftigten immer wieder von unvorhergesehenen Belastungsspitzen*: „Der KUNDE ist halt nicht kontrollierbar. Also sozusagen diese Unwägbarkeit des Faktors KUNDE. […] Wir hatten das in unserem Restaurant, da haben die gesagt: ‚Die sprechen sich alle ab, die kommen irgendwie um 11:30 Uhr alle wie geballt, dann stehen sie alle hier Schlange. Und manchmal ist um 11:30 Uhr noch nichts.‘ Der Kunde als Gestaltungsfaktor sozusagen, der ja ein Eigenleben hat und der nicht immer so will, wie man das vielleicht selber dann mal geplant hat“ (Beschäftigter Betriebsgastronomie).*

#### Eine hohe Interaktionsintensität

umfasst sowohl eine hohe qualitative als auch quantitative Interaktionsanforderung. Während die *quantitative Interaktionsanforderung* die Menge, Geschwindigkeit und Zeit, in der die Interaktionsarbeit zu verrichten ist, beschreibt, bezieht sich die *qualitative Interaktionsanforderung* auf die Komplexität, Schwere und Qualität der zu leistenden Arbeit mit KundInnen. Dabei kann sich die Interaktionsintensität in Abhängigkeit des Tätigkeitsfeldes sehr unterschiedlich darstellen. Während bspw. Beschäftigte in der Systemgastronomie von einer hohen Taktung der Interaktionen mit GästInnen berichteten, erlebten UnternehmensberaterInnen vor allem die Komplexität und Schwere der Interaktionen mit ihren KlientInnen als anstrengend:* „Sie müssen sich VOLL einlassen, ja, das ist die anstrengendste Arbeit, weil sie die Energie des anderen ja total aufnehmen und überlegen müssen, ey, ja, hm, das geht hier jetzt gerade nicht weiter, ne? […] Das ist anstrengend für mich“ (Beschäftigter Unternehmensberatung).*

### Ressourcen auf der Interaktionsebene

Die Ergebnisse der qualitativen Studie verweisen darauf, dass mit der Arbeit an und mit Menschen neben den beschriebenen Stressoren auch vielfältige Ressourcen verbunden sind. So muss die Interaktion zwischen Dienstleistenden und KundInnen nicht nur negativ verlaufen, sondern kann mit verschiedenen positiven Erfahrungen für beide InteraktionspartnerInnen verbunden sein. Auf der Interaktionsebene ließen sich drei Hauptkategorien von Ressourcen identifizieren (Tab. 7 im Anhang).

#### Positive arbeitsbezogene Kundenverhaltensweisen

Nicht nur KollegInnen und Führungskräfte, sondern auch KundInnen können eine Quelle von sozialer Unterstützung sein. So drückte sich ein *partizipatives und kooperatives Verhalten* darin aus, dass KundInnen die Dienstleistenden bei alltäglichen Arbeitsaufgaben unterstützten, indem diese Arbeitsprozesse erleichterten*: „Dann haben wir auch eine Liebe gehabt, die auch immer GEHOLFEN hat – zum Beispiel [die] Getränkerunde [zu übernehmen]“ (Beschäftigte in der stationären Altenpflege). *Zudem unterstützen KundInnen die Dienstleistenden durch *nützliche Informationen *(bspw. vorab recherchierte Artikelnummern zu Produkten) oder durch *wertvolles Feedback *(z. B. konstruktive Verbesserungsvorschläge). Weitergehend zeigte sich die soziale Unterstützung von KundInnen darin, dass sie die erbrachte Dienstleistung *wertschätzten* oder *Verständnis* für die Situation der Beschäftigten aufbrachten. Gemein ist den zuvor dargestellten positiven Verhaltensweisen, dass sie ein Verhalten beschreiben, welches auf der Freiwilligkeit von KundInnen beruht und von solchen Verhaltensweisen abzugrenzen ist, die für die Erbringung der Dienstleistung zwingend erforderlich sind.

#### Positive personenbezogene Kundenverhaltensweisen

KundInnen erleichterten jedoch nicht nur die Arbeit, sondern konnten auch für die Dienstleistenden persönlich eine Bereicherung darstellen. So stellten die Befragten immer wieder die Zusammenarbeit mit *höflichen, netten und freundlichen *KundInnen heraus. Insbesondere in Beschäftigtengruppen, in denen ein hoher und intensiver Kundenkontakt (z. B. Pflege, Unternehmensberatung) bestand, ergaben sich langfristig *freundschaftliche Beziehungen* mit KundInnen. Eng verbunden mit einer reziproken Dienstleistungsbeziehung war die *emotionale Unterstützung*, die die Beschäftigten von KundInnen erhielten (z. B. Mitgefühl oder Ratschläge von KundInnen).

#### Ressourcen aus der interaktiven Tätigkeit

Die Mehrheit der Beschäftigten gab an, dass der primäre Grund ihrer Berufswahl darin liege, mit Menschen arbeiten zu wollen. So erlebten viele Befragten ihren Beruf als persönlich erfüllend: *„es ist halt schon auch ein befriedigendes und erfüllendes Gefühl, wenn ich Menschen helfen kann“ (Beschäftigte in der stationären Altenpflege).* Insbesondere die Zufriedenheit und die Wertschätzung der KundInnen stellte für die Dienstleistenden die Motivation dar, tagtäglich ihren Beruf auszuüben: *„Wenn du ein LACHEN ins Gesicht von irgendeinem zauberst, das ist für mich die Motivation, täglich zur Arbeit zu gehen*“ *(Beschäftigter in der Einzelgastronomie)*. Dabei hoben die Befragten immer wieder hervor, dass sie die Arbeit mit ihren KundInnen in hohem Maße als *sinnstiftend* und *abwechslungsreich *erlebten: *„Meine Arbeit ist per se sinnvoll. Sonst würde ich sie nicht tun. Also der Sinn, der zeigt sich in jeder SEKUNDE eigentlich, ja, warum ich das mache“ (Beschäftigter Unternehmensberatung).*

### Gestaltungsfaktoren auf arbeitsorganisatorischer Ebene

**Figure 5:**
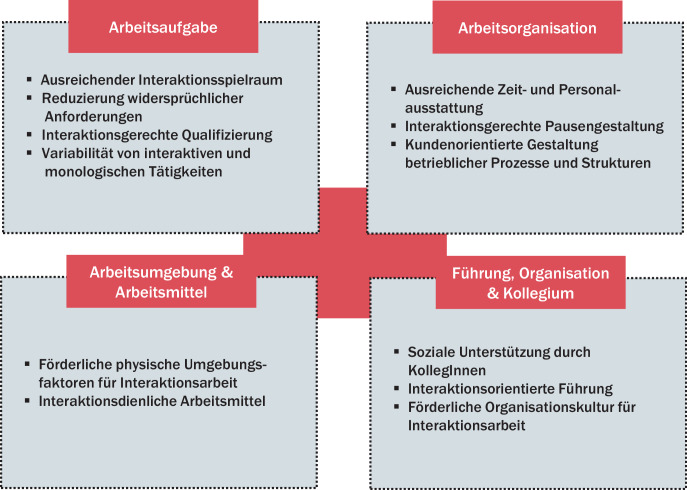

Die Ergebnisse der qualitativen Studie verdeutlichen vor allem die wichtige Rolle der arbeitsorganisatorischen Rahmenbedingungen, unter denen Interaktionsarbeit erbracht wird. Letztere konnten die zu erbringende Interaktionsarbeit potenziell begünstigen oder beeinträchtigen. Folgend werden zentrale arbeitsgestalterische Faktoren identifiziert, systematisiert und diese hinsichtlich ihrer Relevanz für eine menschengerechte Arbeitsgestaltung von Interaktionsarbeit reflektiert (Abb. 5). Auf Basis des Interviewmaterials ließen sich vier übergeordnete Themenfelder branchen- und berufsübergreifender Gestaltungsfaktoren von Interaktionsarbeit identifizieren, die zum Teil mit den in bestehenden Studien diskutierten Faktoren übereinstimmten (Böhle et al. 2015; Hacker et al. 2020; GDA 2018).

#### Gestaltungsfaktoren aus der Arbeitsaufgabe

Es ließen sich vier Gestaltungsfaktoren aus der Arbeitsaufgabe ableiten, die sich durch ihre positive (förderliche) Wirkung auf die Interaktionsarbeit und deren Erbringer auszeichneten (Tab. 8 im Anhang).

1. *Ausreichender Interaktionsspielraum.* Der zur Verfügung stehende Interaktionsspielraum beschreibt, inwieweit Beschäftigte selbst bestimmen können, auf welche Art und Weise die zu leistende Interaktionsarbeit erbracht wird. Konkret bezieht sich der Interaktionsspielraum auf die objektiv vorhandenen und subjektiv wahrgenommenen Wahlmöglichkeiten der Dauer, der Häufigkeit, des Inhalts der Interaktion sowie den gezeigten Gefühlsausdruck. Darüber hinaus umfasst das Konzept Handlungsspielräume bei Entscheidungen, die unmittelbar im Zusammenhang mit der Erbringung der Dienstleistung stehen (z. B. Reklamationsentscheidungen).
2. *Reduzierung widersprüchlicher Anforderungen.* Auf Basis der qualitativen Studie konnten verschiedene Formen von widersprüchlichen Anforderungen identifiziert werden. Widersprüchliche Anforderungen ergaben sich u. a. aus dem Zusammentreffen unterschiedlicher Arbeitsziele. So standen Dienstleistende einerseits vor der Herausforderung, vorgegebene Arbeitsaufgaben (z. B. Disposition von Ware) auszuführen und andererseits KundInnen beraten zu müssen. Die Folge waren wiederkehrende unvorhergesehene Unterbrechungen durch KundInnen.
3. *Interaktionsgerechte Qualifizierung.* Oftmals wurden Fähigkeiten zur Bewältigung der Interaktionsaufgabe vorausgesetzt, ohne dass diese Bestandteile von Qualifizierungsmaßnahmen oder Ausbildungsinhalten waren. Eine Qualifizierung für Interaktionsarbeit zielt dabei auf den Aufbau, den Erhalt und den Ausbau von Fähigkeiten und Fertigkeiten, die zur Bewältigung der Interaktionsaufgabe notwendig sind. Mit dem Konzept wird der Blick darauf gerichtet, dass sich das Arbeitshandeln der Dienstleistenden nicht nur auf sachliche Arbeitsaufgaben begrenzt, sondern jene „Kernaufgaben“ erst durch soziale Kompetenzen ermöglicht werden (z. B. eine gute Emotionsregulation).
4. *Variabilität von interaktiven und monologischen Tätigkeiten. *Insbesondere in Beschäftigtengruppen, in denen ein hoher und intensiver Kundenkontakt bestand, wurde die dauerhafte Interaktion mit KundInnen, PatientInnen und KlientInnen als erschöpfend wahrgenommen. Bei der Aufgabenplanung sollte daher auf eine ausgewogene Verteilung sowohl interaktiver als auch monologischer Tätigkeitsanteile geachtet werden.

#### Gestaltungsfaktoren aus der Arbeitsorganisation

Weitergehend konnten drei Gestaltungsfaktoren aus der Arbeitsorganisation identifiziert werden.

1. *Ausreichende Zeit- und Personalbemessung für Interaktionsarbeit.* Die Beschäftigten betonten immer wieder aufgrund einer zu hohen Arbeitsmenge und einer zu geringen personellen Ausstattung, nicht ausreichend Zeit für die Wünsche und Bedürfnisse der KundInnen, PatientInnen und KlientInnen zu haben. Insbesondere Beschäftigte, die einen hohen Anspruch an ihre eigene Arbeit hatten, erlebten den inneren Rollenkonflikt zwischen dem Grund ihrer Berufswahl und den real vorherrschenden betrieblichen Bedingungen als belastend.
2. *Interaktionsgerechte Pausengestaltung. *Insbesondere in Beschäftigtengruppen, in denen eine hohe Interaktionsintensität bestand, wurde von den Befragten immer wieder die Relevanz interaktionsfreier Pausen hervorgehoben. Eine ausreichende Pausengestaltung ermöglichte es den Beschäftigten, sich in interaktionsfreien Rückzugsräumen von der erbrachten Interaktionsarbeit zu erholen.
3. *Kundenorientierte Gestaltung betrieblicher Prozesse und Strukturen.* Im Rahmen der Interviews zeigte sich, dass schwierige KundInnen häufig erst infolge ungünstiger betrieblicher Rahmenbedingungen „geschaffen“ wurden. Als Ursachen für negatives Kundenverhalten konnten im Rahmen der Interviews lange Wartezeiten, ungenießbare Gerichte, hohe Preise sowie nicht verfügbare oder schlechte Produkte identifiziert werden, die dazu führten, dass KundInnen ihren Unmut gegenüber den Dienstleistenden zum Ausdruck brachten. Umgekehrt zeigte sich, dass eine kundenorientierte Gestaltung betrieblicher Strukturen einen positiven Einfluss auf das Verhalten der KundInnen haben konnte (z. B. ein kulantes Reklamationsmanagement).

#### Gestaltungsfaktoren aus der Arbeitsumgebung und den Arbeitsmitteln

Auf Basis der Interviews ließen sich zwei Gestaltungsfaktoren aus der Arbeitsumgebung und den Arbeitsmitteln ableiten.

1. *Förderliche physische Umgebungsfaktoren für Interaktionsarbeit. *Nicht nur lange Wartezeiten, sondern auch Umgebungsfaktoren wie unangenehm laute Musik, unsaubere Verkaufsräume oder heiße klimatische Bedingungen hatten einen Einfluss auf die Interaktionssituation. So berichten Beschäftigte eines Möbelhauses, dass die im Hintergrund laufende Musik, spielende Kinder oder störende Geräusche von Ausstellungsobjekten (z. B. Musikboxen) die Kommunikation mit KundInnen erschwerten. Zugleich konnte eine förderliche Gestaltung der physischen Umgebung im Bereich des Fallmanagements dazu beitragen, dass KundInnen sich wohlfühlten und eher bereit waren, sich in Beratungsgesprächen zu öffnen.
2. *Interaktionsdienliche Arbeitsmittel. *Bei der Gestaltung von Arbeitsmitteln ist darauf zu achten, dass diese Interaktionsarbeit erleichtern und nicht erschweren. Insbesondere in Zeiten der SARS-COV‑2 Pandemie wurden die vorgeschriebenen Masken und Hygieneschutzwände als hinderlich für die Kommunikation mit den KundInnen wahrgenommen. Gleichwohl konnten technische Hilfsmittel wie Warensuchsysteme VerkäuferInnen dabei unterstützen, typische Kundenfragen (Preis, Verfügbarkeit, Standort des Artikels) schnell und bedürfnisgerecht zu beantworten.

#### Gestaltungsfaktoren aus der Führung, dem Kollegium und der Kultur

Weitergehend konnten drei Gestaltungsfaktoren aus der Führung, dem Kollegium und der Organisationskultur identifiziert werden.

1. *Soziale Unterstützung durch KollegInnen. *In den Interviews wurde immer wieder die Rolle der sozialen Unterstützung von KollegInnen hervorgehoben. Insbesondere bei traumatischen Erfahrungen wie dem Sterben von PatientInnen oder konfliktreichen Auseinandersetzungen mit KundInnen kam den Austauschmöglichkeiten mit KollegInnen eine besondere Stellung zu. Dabei konnten formelle Angebote wie kollegiale Fallberatungen, Supervisionsformate oder Mentorenprogramme helfen, belastende Erlebnisse besser zu verarbeiten. Nicht zu unterschätzen waren dabei informelle Möglichkeiten des kollegialen Austausches (z. B. kurzfristige gemeinsame Pausen), die es ermöglichten, zeitnah über negative Ereignisse mit KundInnen zu sprechen.
2. *Interaktionsorientierte Führung. *Als ein Schlüsselfaktor in der Gestaltung von Interaktionsarbeit stellte sich die Einflussnahme von Führungskräften bei der Erfüllung der Interaktionsaufgabe heraus. Dabei kam der Führungskraft einerseits eine wichtige Verantwortung bei der Motivation von Mitarbeitenden und andererseits eine schützende Rolle zu. So hatten Führungskräfte in den kritischen Auseinandersetzungen die Aufgabe, KundInnen Grenzen aufzuzeigen oder in drastischen Fällen sogar Hausverbote auszusprechen.
3. *Förderliche Organisationskultur für Interaktionsarbeit. *Als eine förderliche Organisationskultur für Interaktionsarbeit beschrieben die Beschäftigten eine Kultur, die von wechselseitigem Vertrauen geprägt ist, eine hierarchieübergreifende Kommunikation sowie Partizipation ermöglicht und organisationale Gerechtigkeit garantiert. Als ungerecht erlebten Beschäftigte, wenn diese versuchten, die von der Organisation vorgegebenen Reklamationsvorschriften durchzusetzen und bei Hilferufen die Vorgesetzten ihnen in den Rücken fielen.

## Diskussion

Ziel der Studie war es, interaktionsbezogene Stressoren und Ressourcen zu identifizieren, zu systematisieren und diese hinsichtlich ihrer Relevanz für eine menschengerechte Arbeitsgestaltung von Interaktionsarbeit zu reflektieren. Damit leistet der vorliegende Artikel nicht nur einen Beitrag, interaktionsbezogene Stressoren und Ressourcen tiefergreifend zu erforschen, sondern schafft ebenfalls einen konzeptionellen Rahmen, um Interaktionsarbeit im Sinne einer prospektiven Arbeitsgestaltung menschengerecht gestalten zu können.

Die Ergebnisse der Literaturanalyse zeigen, dass in der bestehenden Debatte um den Forschungsgegenstand der Interaktionsarbeit bislang nur wenige systematische Erkenntnisse zu den arbeitsorganisatorischen Rahmenbedingungen, unter denen Interaktionsarbeit erbracht wird, bestanden (Böhle und Weihrich 2020). Der vorliegende Artikel verdeutlicht mit seinen Ergebnissen, dass oftmals Rahmenbedingungen darüber entscheiden, inwieweit Dienstleistende ihre Arbeit mit KundInnen, PatientInnen oder KlientInnen als Stressor oder Ressource wahrnehmen. Mit der Unterscheidung von Stressoren und Ressourcen auf der Interaktionsebene sowie gestaltungsrelevanter Faktoren auf der arbeitsorganisatorischen Ebene hebt der Artikel hervor, dass das untersuchte Themenfeld aus einer Multi-Level-Perspektive heraus betrachtet werden sollte. In Rückbezug zu dem vorgestellten JD‑R Modell, welches als theoretische Grundlage für Identifizierung interaktionsbezogener Stressoren und Ressourcen diente, zeigt sich, dass eine Multi-Level-Perspektive bislang weitgehend außer Acht gelassen wurde. So kommen Bakker und Demerouti (2017) in ihrem Artikel zu offenen Forschungsbedarfen des JD‑R Modells zu dem Schluss: „We […] encourage researchers to integrate multiple levels in their research using JD‑R theory“ (Bakker und Demorouti 2017, S. 281). Die Berücksichtigung einer Multi-Level-Perspektive eröffnete die Möglichkeit, das komplexe Zusammenwirken arbeitsorganisatorischer Rahmenbedingungen und Stressoren und Ressourcen auf der Interaktionsebene aufzuzeigen. So verweisen die Ergebnisse der qualitativen Studie darauf, dass schwierige KundInnen häufig erst in Folge ungünstiger Rahmenbedingungen geschaffen werden, wenn diese bspw. in langen Schlangen warten müssen und infolgedessen ihren Frust gegenüber den Dienstleistenden kanalisierten. Lange Wartezeiten der KundInnen resultierten wiederum daraus, dass bei der Personaleinsatzplanung zu wenig Zeit für die Kundenberatung berücksichtigt wurde. Das dargestellte Beispiel veranschaulicht, dass arbeitsorganisatorische Rahmenbedingungen einen direkten Einfluss auf die zu leistende Interaktionsarbeit nehmen und je nach Art und Ausprägung sowohl mit positiven als auch negativen Folgen für die Beschäftigten, die KundInnen und die Organisation verbunden sein konnten. Damit verdeutlicht der vorliegende Artikel, abweichend von der bestehenden Perspektive in der Forschung „der Kunde sei nicht gestaltbar“, dass Organisationen durchaus mittels gezielter arbeitsgestalterischer Maßnahmen einen Einfluss auf das Verhalten von KundInnen nehmen können. In Zukunft gilt es daher, die förderlichen und beeinträchtigenden Wirkungen arbeitsorganisatorischer Rahmenbedingungen in quantitativen Studien tiefergreifend zu erforschen.

Weitergehend zeigen die Ergebnisse der Literaturanalyse, dass bislang nur vereinzelt Studien bestehen, die sich mit den positiven Seiten von Interaktionsarbeit und deren gesundheits- und motivationsfördernder Wirkung auf Beschäftigte auseinandersetzen. Der vorliegende Artikel leistet einen Beitrag, die bestehende Debatte über den Forschungsgegenstand der Interaktionsarbeit um eine Ressourcenperspektive zu erweitern. Die Ergebnisse der qualitativen Studie verdeutlichen, dass KundInnen nicht nur die Rolle von „Trottelkunden“ (Wirtz und Lovelock 2021), „Problemkunden“ (Bitner et al. 1994) oder auch „Kunden aus der Hölle“ (Withiam 1998) zukommt, sondern für Dienstleistende vielfach eine persönliche Ressource darstellen können. Dabei verweisen die Ergebnisse darauf, dass KundInnen über ihre zu erwartende Rolle hinaus ein Extra-Rollenverhalten (Bettencourt 1997) zeigen, indem sie Dienstleistenden bei der Erbringung ihrer Arbeitsaufgaben halfen, wertvolles Feedback gaben oder diese emotional unterstützten. Jene positiven Kundeninteraktionen stellen vielfach den Grund dar, warum Dienstleistende ihre Tätigkeit als abwechslungsreich und persönlich erfüllend erlebten. Damit verweisen die Ergebnisse abweichend von der bisherigen pathogenetischen Betrachtung in der Literatur auf die mögliche positive Wirkung von Interaktionsarbeit für die Gesundheit und das Befinden von Beschäftigten. Zukünftige Forschung sollte in Rückbezug zu den im JD‑R Modell postulierten „motivational process“ und „health impairment process“ in Längsschnittstudien untersuchen, wie sich interaktionsbezogene Ressourcen und Stressoren in ihren kurz-, mittel- bis hin zu langfristigen Wirkungseffekten sowohl positiv als auch negativ auf die Gesundheit von Beschäftigten auswirken. Zudem sollten neben Antezedenzien von positivem und negativem Kundenverhalten ebenfalls Crossover-Effekte in dyadischen Studiendesigns zwischen KundInnen und Dienstleistenden betrachtet werden. Damit würde es möglich werden, das komplexe Zusammenwirken des Verhaltens von KundInnen und Dienstleistenden tiefergreifend zu erforschen.

### Limitationen

Zunächst muss bei der Interpretation der Ergebnisse der Literaturanalyse berücksichtigt werden, dass das eingeschränkt systematische Vorgehen sowie die Auswahl von Datenbanken, überwiegend psychologischer Journals, möglicherweise dazu führte, dass der Forschungsstand zu interaktionsbezogenen Stressoren und Ressourcen nicht erschöpfend dargestellt wurde. In Zukunft sollten daher Studien weitergehende systematische Literaturanalysen durchführen, wobei die Ergebnisse der qualitativen Studie erste Impulse für konkrete Suchstrings bieten könnten. Darüber hinaus ist kritisch zu betrachten, dass die Interviews sowohl virtuell als auch physisch vor Ort in den Organisationen durchgeführt wurden. Das Medium der Interviewführung könnte insofern einen Einfluss auf die Ergebnisse der qualitativen Studie haben, da Befragte in den Interviews vor Ort deutlich offener über kritische Herausforderungen (z. B. Umgang mit Tod) ihrer Zusammenarbeit mit KundInnen, PatientInnen und KlientInnen berichten. Zudem ist bei der Interpretation der Ergebnisse der Interviews und teilnehmenden Beobachtungen zu berücksichtigen, dass der Durchführungszeitraum die SARS-COV‑2 Pandemie miteinschloss. Dies ist insofern bedeutsam, da aufgrund der vorgeschriebenen Hygienemaßnahmen Beschäftigte deutlich häufiger mit negativem Kundenverhalten konfrontiert waren und betriebliche Abläufe in den teilnehmenden Organisationen verändert waren. Weiterhin gilt es zudem zu bedenken, dass im Rahmen der Interviews lediglich nach den Einschätzungen der Dienstleistenden gefragt, die Perspektive von KundInnen jedoch außer Acht gelassen wurde. Um Ursachen von negativem Kundenverhalten tiefergreifend zu erforschen und gezielte verhältnispräventive Maßnahmen zur Gestaltung von Interaktionsarbeit ableiten zu können, bedarf es weiterer Studien, in denen KundInnen befragt werden. Ferner war es aufgrund der SARS-COV‑2 Pandemie nicht möglich, Beobachtungen in allen Beschäftigtengruppen durchzuführen. So muss bei der Einordnung der Ergebnisse berücksichtigt werden, dass in den Beschäftigtengruppen der Polizei und des Fallmanagements Rückschlüsse auf arbeitsorganisatorische Rahmenbedingungen nur auf Basis der subjektiven Einschätzungen der Befragten gezogen werden können.

## Schlussfolgerungen für die betriebliche Praxis

Die Ergebnisse des vorliegenden Beitrags verdeutlichen, dass in der betrieblichen Praxis zunehmend verhältnispräventive Maßnahmen zur Gestaltung von Interaktionsarbeit in den Blick zu nehmen sind. So sollten Maßnahmen ergriffen werden, die darauf abzielen, die Auftretenswahrscheinlichkeit von negativem Kundenverhalten schon in der Entstehung zu verringern. Beispielhaft konnten hierbei im Rahmen der qualitativen Studie Maßnahmen wie die Implementierung eines kulanten Reklamations- und Beschwerdemanagements oder ein verbessertes Management der Produktqualität angeführt werden. Dabei könnte insbesondere der Gefährdungsbeurteilung psychischer Belastung eine zentrale Bedeutung zukommen, interaktionsspezifische Gefährdungen zu erfassen, zielgerichtete Maßnahmen abzuleiten und deren Wirksamkeit zu überprüfen. Mit der Identifizierung relevanter Gestaltungsfaktoren wurden erste Themeninhalte für die Gefährdungsbeurteilung von Interaktionsarbeit aufgezeigt. In Zukunft sind vor allem geeignete Methoden zu entwickeln, die die dargestellten interaktionsspezifischen Gefährdungen betriebsspezifisch erfassen.

## Anhang

**Table 2:**

Spezifizierung	Suchbegriffe
*Interaktionsbezogene Stressoren*	Interaktionsbezogene Stressoren, Stressoren in der Interaktionsarbeit, Soziale Stressoren Arbeitswelt, Psychische Belastung Interaktionsarbeit, Stressor Kunde, Negatives Kundenveralten, customer-related social stressors, customer unfriendliness, customer mistreatment, unfair behaviour, customer incivility, customer aggression, customer violence, customer hostility, jay customer behaviour
*Interaktionsbezogene Ressourcen*	Interaktionsbezogene Ressourcen, Ressourcen in der Interaktionsarbeit, Soziale Ressourcen Arbeitswelt, Ressourcen Kunde, Positives Kundenverhalten, Soziale Unterstützung Kunde, customer ressource, customer friendliness, customer support, customer cooperation, customer participation, customer voluntary behaviour, customer citizenship behaviour

**Table 3:**

Kategorie	Beobachtungsaspekte
*Tätigkeitsbeschreibung*	Welche Tätigkeiten führt der Beschäftigte aus?
	Wie wichtig ist Interaktionsarbeit für das Gelingen der Tätigkeit?
	Wie stehen interaktive und nicht-interaktive Tätigkeitsanteile im Zusammenhang?
*Interaktionscharakteristika*	Wie lang dauert die Interaktion pro Kunde?
	Mit wie vielen Kunden interagiert der Beschäftigte (Beschäftigte pro Stunde [ggf. andere Zeiteinheit, z. B. pro Tag]; Taktung von Interaktionen)?
	Wer ist der Initiator der Interaktion (Kunde oder Beschäftigter)?
	Wie ist die Richtung der Interaktion (eher einseitig oder wechselseitig)?
	Was ist Inhalt der Interaktion?
	Wie ist der ungefähre Anteil der Interaktionsarbeit an der gesamten Arbeit?
	Welche Art der Interaktionsbeziehung liegt vor (Encounter-Laufkunden vs. Relationship-Stammkunden)?
	Wie ist das Ausmaß der Standardisierung der Interaktion (laufen Interaktionen ähnlich/gleich ab; welche Elemente ähneln bzw. unterscheiden sich)?
	Wie stehen die externe (z. B. KundInnen, PatientInnen und KlientInnen) und interne Interaktionsarbeit (z. B. Führungskräfte, KollegInnen) im Zusammenhang?
*Verhalten der Interaktionspartner*	Wer sind die Kunden, die mit den Beschäftigten in Interaktion treten?
	Wie verhält sich der Beschäftigte(r) gegenüber dem Kunden?
	Wie verhalten sich Kunde(n) gegenüber Beschäftigten?
	Was sind Herausforderungen im Umgang mit Kunden (z. B. schwierige Kunden, Meinungsverschiedenheiten, Konflikte)?
	Wie geht der Beschäftigte mit den beschriebenen Herausforderungen um?
	Wie manifestieren sich negative Verhaltensweisen von Kunden?
	Was sind Ursachen und Hintergründe für negatives Kundenverhalten (z. B. lange Wartezeiten)?
	Was sind positive Verhaltensweisen von Kunden? Wie unterstützen die Kunden Beschäftigte bei ihrer Arbeit?
	Was sind Ursachen und Hintergründe für positives Kundenverhalten (z. B. gutes Reklamationsmanagement)?
*Arbeitsorganisatorische Rahmenbedingungen von Interaktionsarbeit*	Unter welchen Rahmenbedingungen findet die zu leistende Interaktionsarbeit statt?
	Was sind Rahmenbedingungen, die die zu leistende Interaktionsarbeit erleichtern?
	Was sind Rahmenbedingungen, die die zu leistende Interaktionsarbeit erschweren?
	Gibt es Merkmale der Arbeit, die für Beschäftigte schwierig sind miteinander zu vereinbaren (Widersprüchliche Anforderungen)? Wenn ja, wie gehen die Beschäftigten damit um?
	Gibt es Arbeitsprozesse, die die Beschäftigten bei Ihrer Arbeit mit Ihren Kunden behindern?
	Was sind Maßnahmen, die von der Organisation ergriffen werden, um schwierigem Kundenverhalten vorzubeugen?
	Was sind Arbeitsmittel, die bei der Arbeit mit Kunden verwendet werden? Wie nehmen Arbeitsmittel einen Einfluss auf die zu leistende Interaktionsarbeit?
	In welcher Arbeitsumgebung findet die Interaktion mit dem Kunden statt?
	Werden Beschäftigte unterbrochen? Was sind Quellen von Unterbrechungen? Wie wirken sich Unterbrechungen auf die zu leistende Interaktionsarbeit aus?

**Table 4:**

AutorInnen (Jahr)	Branchen	Vergl. Std.	Studiendesign	Stichprobe	Betrachtete Konstrukte	Ergebnisse
Bitner et al. (1994)	Hotels, Restaurants, Fluggesellschaft	Nein	Critical-Incident Technik	*N* = 781 Incidents *n* = 58 Hotels *n* = 152 Restaurants *n* = 4 Fluglinien	**UV:** Drunkenness, verbal and physical abuse, breaking company policies or laws, uncooperative customers **AV:** Customer dissatisfaction	Die Ergebnisse deuten darauf hin, dass Kundenunzufriedenheit auf das Fehlverhalten der Kunden selbst rückzuführen ist
Böhle et al. (2015)	Einzelhandel, Gastronomie, Arbeitsverwaltung, Krankenpflege	Ja	Qualitative Studie	*N* = 48 *n* = 16 Einzelhandel *n* = 6 Gastronomie *n* = 16 Arbeitsverwaltung *n* = 10 Pflege	Überprüfung der Kriterien der humanen Arbeitsgestaltung für Interaktionsarbeit	Die Ergebnisse zeigen, dass schwierige Kunden oftmals erst durch die Dienstleistungsorganisation geschaffen werden. Böhle et al. (2015) führen schwieriges Kundenverhalten auf Faktoren wie eine fehlende Preisauszeichnungen oder eine unlogische, aber verkaufsförderliche Anordnung der Waren zurück
Cantu und Thomas (2020)	Krankenhaus	Nein	Quantitative Befragung	*n* = 39 Beschäftigte des Krankenhauses	**UV:** Critical Incidents (CI) **AV: **Hospital anxiety and depression, Professional quality of life (ProQOL)	Die Beschäftigten nannten als kritische Vorfälle bei ihrer Arbeit den Umgang mit schwerkranken Kindern, Massenunfälle sowie das Sterben von Patienten – die kritischen Vorfälle ereigneten sich in der Regel einmal pro Woche
Dormann und Zapf (2004)	Tourismus, Einzelhandel	Nein	Querschnittsanalyse/Regressionsanalyse	*N* = 591 *n* = 312 Flugbegleiter *n* = 88 Schuhverkäufer *n* = 191 Beschäftigte im Reisebüro	**UV: **Customer-Related Social Stressors **AV:** Burnout	Die vier Subskalen der CSS-Skala waren mit den drei Subfacetten des Burnout-Syndroms über verschiedene Kontrollvariablen (Branche, Alter, Geschlecht usw.) assoziiert
Dormann et al. (2017)	Polizei	Nein	Längsschnittstudie	*n* = 332 Polizeibeamte	**UV:** Customer-Related Social Stressors, Age, Gender **AV: **Police officers’ negative and positive affect	Die vier Subskalen der CSS-Skala waren mit einem negativen Affekt der Polizeibeamten assoziiert, wobei dieser Zusammenhang durch das Geschlecht und das Alter moderiert wurde. So waren das Erleben von kundenbezogenen Stressoren bei älteren sowie weiblichen Polizeibeamten tendenziell mit einem schwächeren Anstieg des negativen Affekts als bei jüngeren bzw. männlichen Beamten verbunden
Dudenhöffer und Dormann (2013)	Öffentlicher Dienst	Nein	Längsschnittstudie/Tagebuchstudie	*n* = 106 Beschäftigte im öffentlichen Dienst	**UV:** Daily, Periodic, and General Customer-Related Social Stressor **AV: **Sort-term (across a day) and mid-term (across 2 weeks) affective reactions to perceived CSS	Soziale kundenbezogene Stressoren (CSS) können in ihrer kurzfristigen bis mittelfristigen Wirkung zu negativen affektiven Reaktionen wie Beunruhigung, Aufregung, Reizbarkeit, Nervosität oder Angst bei den Beschäftigten führen
Dudenhoffer und Dormann (2015)	Flugbegleiter, Schuhverkäufer, Pflegekräfte, Bankangestellte, Versicherungsangestellte, Secruity-Angestellte, Lehrer, Police Officer	Ja	Metaanalyse	Mitarbeitende N = 4199 (Verschiedene Beschäftigte in Dienstleistungsberufen)	**UV: **Customer-Related Social Stressors **AV**: Emotionale Dissonanz, Burnout, Arbeitszufriedenheit	Soziale kundenbezogene Stressoren waren positiv mit den Subfacetten des Burnout-Syndroms und negativ mit der Arbeitszufriedenheit der Dienstleistenden verbunden
Fisk und Neville (2011)	Gastronomie	Nein	Interviews	Mitarbeitende *n* = 56 Beschäftigte in der Gastronomie	**UV:** customer entitlement as a form of micro aggression **AV: **physiological arousal, negative affect, burnout, and feelings of dehumanization as a result of dealing with these patrons	Die Ergebnisse zeigten, dass sich das anspruchsvolle Verhalten von Gästen negativ auf die Servicekräfte auswirkte. Die Beschäftigten berichteten von physiologischer Erregung, einem negativen Affekt, Burnout-Symptomen und dem Gefühl der Entmenschlichung als Folge des Umgangs mit diesen Gästen
Gettman und Gelfand (2007)	Einzelhandel	Nein	Qualitative Interviews und quantitative Befragung	Mitarbeitende Studie 1 *n* = 394 (Beschäftigte im Büro – Pilotstudie) Studie 2 *n* = 2519 Beschäftigte im Einzelhandel	**UV: **Client Gender Context, Perceived Client Power, Client Accountability, Service Pressure Climate **AV:** Customer Sexual Harassment Job Satisfaction, Health Satisfaction Psychological Distress	Die Ergebnisse zeigten, dass sexuelle Belästigung mit einer geringeren Arbeitszufriedenheit der befragten weiblichen Dienstleistenden verbunden war
Grandey et al. (2004)	Call Center	Nein	Interviews, quantitative Befragung	*n* = 198 Beschäftigte im Callcenter	**UV:** customer aggression **AV:** emotional exhaustion, absence	Beschäftigte berichteten, dass sie im Durchschnitt 10 Mal pro Tag mit verbalen Aggressionen von Beschäftigten konfrontiert waren. Die Häufigkeit der verbalen Aggression von Kunden stand im Zusammenhang mit einem erhöhten Erleben von emotionaler Erschöpfung
Harris und Reynolds (2004)	Hotels und Restaurants	Nein	Critical-Incident Technik	*N* = 106 *n* = 29 Kunden *n* = 46 Mitarbeiter *n* = 31 Führungskräfte	Exploratives Design um Jaycustomer behaviour zu untersuchen	Die Studie zeigt acht grundlegende Arten von Jaycustomer behaviour: 1) Beschwerdeschreiber, 2) nicht zufriedenzustellende Kunden, 3) Kunden, die das Eigentum beschädigen, 4) Kunden, die sich selbst als „Dienstleistende“ sehen, 5) rachsüchtige Kunden, 6) verbal aggressive Kunden, 7) physisch aggressive Kunden, 8) sexuell übergriffige Kunden
Li und Zhou (2013)	Call Center	Nein	Quantitative Befragung	*n* = 1112 Beschäftigte im Callcenter	**UV: **Customer verbal aggression **AV: **turnover intensions, Emotional Exhaustion, perceived organisational support	Die Ergebnisse veranschaulichen, dass verbale Aggression die Kündigungsabsicht von Dienstleistenden vorhersagt. Dabei medierte die emotionale Erschöpfung der Beschäftigten den Zusammenhang zwischen verbaler Kundenaggression und der Kündigungsabsicht
Harris und Reynolds (2003)	Hotels und Restaurants	Nein	Interviews	*N* = 106 *n* = 29 Kunden *n* = 46 Mitarbeiter *n* = 31 Führungskräfte	**UV: **dysfunctional customer behaviours **AV: 1) Consequences for Employees:** – Long-Term Psychological Consequences – Short-term Emotional Effects – Behavioural Effects **2) Consequences for Customers:** – Domino Effects – Spoilt Consumption Effects **3) Consequences for Organisations:** – Indirect Financial Costs – Direct Financial Costs	Die Autoren entwickeln ein Rahmenmodell zur Beschreibung der Auswirkungen von dysfunktionalem Kundenverhalten auf die Mitarbeiter, Kunden und Organisationen. Die Ergebnisse zeigen, dass von den 46 befragten Beschäftigten 8 Symptome einer psychischen Schädigung aufwiesen, die auf dysfunktionales Kundenverhalten zurückzuführen waren. 93 % der befragten Mitarbeiter gaben an, dass dysfunktionales Kundenverhalten ihr emotionales Befinden negativ beeinflusse. 80 % der männlichen Dienstleistenden berichteten von Konfrontationen mit physischen Aggressionen der Kunden
Reynolds und Harris (2006)	Restaurants	Nein	Interviews	*N* = 85 *n* = 64 Mitarbeiter *n* = 21 Manager	**UV:** Deviant customer behaviour **AV: **Frontline Employee tactics	Die Autoren identifizierten 15 Bewältigungsstrategien von Beschäftigten im Umgang mit Kunden, die sich schlecht benahmen (u. a. Mentale Vorbereitung für die Arbeit, Konsum von Drogen, Ignorieren von schwierigen Kunden, Ausnutzen der eigenen sexuellen Attraktivität, Bestechen von Kunden, mit Kollegen reden, Individuelle Isolation, Rachegelüste der Beschäftigten …)
Reynolds und Harris (2009)	Hotels, Restaurants	Nein	Quantitative Befragung	*n* = 384 befragte Kunden	**UV:** Disaffection with service, service escape, Psychological obstructionism **AV:** severity of dysfunctional customer behaviour	Die Ergebnisse zeigen, dass die Unzufriedenheit der Kunden mit dem Service mit einem dysfunktionalen Kundenverhalten in Zusammenhang steht
Sliter und Jex (2012)	Bankangestellte	Nein	Quantitative Befragung	*n* = 120 Bankangestellte	**UV: **Customer Incivility **AV: **Absenteeism, Tardiness, Sales Performance	Die Autoren berichten im Zuge ihrer bei Bankangestellten durchgeführten Befragung, dass die Interaktion mit unhöflichen Kunden sowohl eine geringere Verkaufsleistung als auch eine Zunahme der Ausfalltage der Beschäftigten zur Folge hatte. Die Autoren führen die geringere Verkaufsleistung darauf zurück, dass das unhöfliche Verhalten der Kunden als Auslöser für ein abnehmendes Engagement und eine mangelnde Leistungsbereitschaft der Dienstleistenden gesehen werden kann
Sliter et al. (2010)	Bankangestellte	Nein	Quantitative Befragung	*n* = 120 Bankangestellte	**UV:** customer incivility, emotional labour **AV:** emotional exhaustion, ratings of customer service quality	Die Befragungsdaten zeigten, dass unhöfliches Kundenverhalten positiv mit der emotionalen Erschöpfung und negativ mit der subjektiven Bewertung der Leistung von Dienstleistenden zusammenhängt
Vanheule et al. (2008)	Sicherheitsbranche	Nein	Quantitative Befragung	*n* = 530 Sicherheitsleute	**UV:** Critical Incidents (CI) **AV:** Burnout	Zu den von den Sicherheitsfachkräften genannten kritischen Vorfällen zählten Körperverletzungen, Morddrohungen (verbal, körperlich, mit Messern und mit vorgehaltener Waffe), die Konfrontation mit Mord, Selbstmord, schweren Verletzungen und Tod infolge von Unfällen oder Überfällen. Die berichteten kritische Ereignisse waren mit den Subfacetten des Burnout-Syndroms assoziiert
Walsh (2011)	Beschäftigte verschiedener Dienstleistungsberufe	Nein	Quantitative Befragung	*n* = 219 Servicemitarbeitende	**UV: **Customer Unfriendliness **AV: **Role Ambiguity, Distance Seeking, Job Satisfaction, Intention to Quit	Die Ergebnisse deuten darauf hin, dass die wahrgenommene Unfreundlichkeit von Kunden einen indirekten und direkten Einfluss auf die Arbeitszufriedenheit der Beschäftigten hat, was wiederum einen positiven Einfluss auf die Kündigungsabsicht der Beschäftigten hatte
Wegge et al. (2007)	Call Center	Nein	Experimentelles Studiendesign	*n* = 96 Beschäftigte im Callcenter	**UV: **Bedingung 1: phone, pc-videoconference or pc-videoconference, Bedingung 2: half of the customers were friendly whereas the other half were rude **AV: **levels of immunoglobulin A (IgA)	Es wurde festgestellt, dass unfreundliches Kundenverhalten zu einer höheren Belastung und einer geringeren Gesprächsleistung der Beschäftigten führte als freundliches Kundenverhalten. Zeitdruck erhöhte die IgA-Werte und verringerte die Gesprächszeit mit den Kunden
Berry und Seiders (2008)	Business-to-business Dienstleistungen	Nein	Review	*n* = keine Angabe (Führungskräfte im Bereich der Verbraucherdienstleistungen)	Analyse verschiedener Formen von unfairem Kundenverhalten	Kunden nutzen die Tatsache aus „immer im Recht“ zu sein, um ungerechtfertigte Privilegien und Entschädigungen gegenüber den Dienstleistenden einzufordern. Im Zuge ihrer Studie identifizieren die Autoren fünf Typen von unfairen Kunden: Blamers, Rule Brakers, Opportunists, Returnaholics
Bell und Luddington (2006)	Einzelhandel	Nein	Regressionsanalyse	*n* = 432 Beschäftigte im Einzelhandel	**UV**: service personnel commitment **AV 1**: customer complaints **AV 2**: positive/negative affectivity	Kundenbeschwerden wirken sich negativ auf das Engagement der Dienstleistenden aus und führen zu Rollenkonflikten, wobei dieser Zusammenhang von dem positiven affektiven Erleben des Dienstleistenden moderiert wurde. Entgegen der Annahmen zeigte sich, dass ein hohes Maß an negativer Affektivität auch die negative Beziehung zwischen Beschwerden und Engagement für den Kundenservice verringerte
Gong et al. (2021)	Hotelkette	Nein	Qualitative Studie (face-to-face interview)	*n* = 25 Beschäftigte im Hotel	**UV: **three different customer interactional injustice (CII) categories (behaviourally offensive, verbally abusive, or overly self-indulgent customers) **AV:** employees’ emotion regulation strategies	Die Ergebnisse zeigen, dass sich die Regulierungsstrategien der Angestellten je nach Ausprägung des ungerechten Verhaltens (CII) unterschieden. Während die Angestellten Problemlösungsstrategien nutzen, wenn sie mit übermäßig selbstverliebten Kunden konfrontiert waren, wurden Vermeidungsstrategien bei verhaltensauffälligen Kunden angewandt. Bei verbal beleidigenden Kunden grübelten die Beschäftigten oftmals über die Erlebnisse im Nachgang
Kim und Baker (2020)	Einzelhandel	Nein	Qualitative Befragung	*n* = 404 Beschäftigte bei Amazon und Mechanical Turk	**UV 1:** Service recovery aimed at other customer: good vs. poor **UV2: **Legitimacy of complaining behaviour of other customers: legitimate vs. illegitimate **UV3:** Emotional expression: aggressive vs. calm **AV:** customers who witness the complaints’ behavioural reactions (revisit intention, tipping behaviour, intention to complain)	Wenn Kunden illegitime Beschwerden anderer Kunden wahrnehmen, beeinflusst das deren Verhalten und deren Reaktionen. So zeigen die Ergebnisse, dass die illegitimen Beschwerden einen signifikanten Haupteffekt auf das Trinkgeldverhalten und die Wiederbesuchsabsicht, nicht aber auf die Beschwerdeabsicht der Kunden hatten
Kang und Gong (2019)	Schönheitssalons	Nein	Strukturgleichungsmodell	*n* = 312 Arbeitnehmende	**UV 1: **Verbal abuse **UV 2:** Disproportionate demand **UV 3: **Illegitimate complaint **AV 1/UV4:** emotional exhaustion **AV 2: **employee withdrawal	Die Ergebnisse zeigen, dass verbale Beschimpfungen, unverhältnismäßige Forderungen und unberechtigte Beschwerden einen signifikanten Einfluss auf die emotionale Erschöpfung von Beschäftigten hatten
Walker et al. (2017)	Versicherungsunternehmen	Nein	Mehrstufige, multisource, mixed-method Feldstudie zu Kundenservice-Events	*n* = 434 Events	**UV 1**: aggressive words **UV 2**: person pronoun use **UV 3**: interruptions **UV 4**: positive Emotion words **AV:** employee incivility	Mitarbeiter im Kundendienst neigen dazu, auf unhöfliches Verhalten von Kunden negativ zu reagieren, indem sie im Gegenzug ebenfalls mit Unhöflichkeit reagieren. Der positive Zusammenhang zwischen verbalen Aggressionen der Kunden und Unhöflichkeit der Mitarbeiter war ausgeprägter, wenn die verbale Aggression der Kunden die Du-Form enthielten („Dutzen“ des Dienstleistenden). Mitarbeiter verhielten sich umso unhöflicher, wenn verbale Aggressionen von Kunden von mehr Unterbrechungen begleitet wurden
Linley und Joseph (2006)	Britisches Katastrophenschutzunternehmen	Nein	Querschnittsanalyse/Längsschnittstudie	*n* = 56 Beschäftigte eines Katastrophenschutzunternehmens	**UV**: traumatic stress (Occupational death exposure **AV**: Posttraumatic growth, Positive changes, Negative changes	Die Studie untersuchte die berufsbedingte Konfrontation mit dem Tod, die Einstellung zum Tod, subjektive Bewertungen, Intrusionen, Vermeidungsverhalten sowie deren Zusammenhang mit positiven und negativen psychologischen Veränderungen bei Beschäftigten im Katastrophenschutz. Je größer das Erleben von Angst, Schrecken und Hilflosigkeit und je größer das Ausmaß der Intrusionen war, desto mehr posttraumatischen Stress berichteten die Beschäftigten. Gleichermaßen zeigte sich, dass je mehr die Beschäftigten subjektive Gefühle der Angst, des Entsetzens und der Hilflosigkeit erlebten, je mehr sie es vermieden, über ihre berufliche Konfrontation mit dem Tod nachzudenken, desto eher berichteten die Beschäftigten negative Veränderungen im Stresserleben
Wegge et al. (2012)	Call Center	Nein	Experimentelles Studiendesign	*n* = 96 Beschäftigte im Callcenter	**UV: **Bedingung 1: phone, pc-videoconference or pc-videoconference, Bedingung 2: half of the customers were friendly whereas the other half were rude **AV:** positive und negative Emotionen, Emotionale Dissonanz, Persönliche Identität, Organisationale Identifikation	Beschäftigte, die mit höflichen Kunden interagierten (Kunden lobten sowohl die Qualität des Produkts als auch die ausgezeichnete Beratung des Dienstleistenden), erleben mehr positive Emotionen und weniger emotionale Dissonanz im Vergleich zu Beschäftigten, die mit unfreundlichen Kunden interagierten (Kunden beschwerten sich über die Qualität des Produkts und beschimpften den Beschäftigten als inkompetent)

**Table 5:**

AutorInnen (Jahr)	Branchen	Vergl. Std.	Studiendesign	Stichprobe	Betrachtete Konstrukte	Ergebnisse
Groth (2005)	Zufallsstichprobe von Kunden – keine spezifische Branche	Nein	Querschnittsanalyse, Regressionsanalyse	*n* = 400	**UV:** customer socialization, customer satisfaction **AV: **Customer coproduction, citizenship behaviours (recommendations, helping customers, providing feedback)	Groth (2005) unterteilen das gezeigte kooperative Verhalten von Dienstleistenden und Kunden hinsichtlich dem erwarteten „Customer coproduction behaviour“ und dem freiwillig gezeigten Kundenverhalten „customer citizenship behaviour“. Beide Arten des Kundenverhaltens hängen mit unterschiedlichen Prädiktoren zusammen. Während das geforderte „Customer coproduction behavior“ stärker durch die Kundensozialisation und die Werte der Organisation vorhergesagt wird, ist das „customer citizenship behaviour“ stärker mit der Kundenzufriedenheit assoziiert
Kim und Yoon (2012)	Einzelhandel	Nein	Querschnittsanalyse, Regressionsanalyse	*n* = 117	**UV 1**: Customer’s Personality Traits **UV 2: **Employee’s Display of Emotions **UV 3/AV 1:** Customer’s Display of Emotions **AV 2:** Employee’s Mood	Kim und Yoon (2012) können in ihrer im Einzelhandel durchgeführten Studie bestätigen, dass die positiven Emotionen von Kunden zu einer positiven Stimmung der Dienstleistenden führen. Die Ergebnisse verdeutlichen, dass die positiven Emotionen des Kunden die Beziehung zwischen den positiven Emotionen des Mitarbeiters und dessen positiven Stimmung vermitteln. Darüber hinaus moderieren die Persönlichkeitsmerkmale des Kunden diese Beziehung
Loera et al. (2016)	Kindergärten und Vorschulen	Nein	Deskriptive Analyse, Reliabilität und explorative Faktorenanalyse	Studie 1: *n* = 105 Studie 2: *n* = 304 Studie 3: *n* = 296	**UV**: Soziale Unterstützung durch den Kunden **AV:** Burnout (Emotional Exhaustion, Depersonalization, Personal accomplishment), Arbeitszufriedenheit	Die soziale Unterstützung von Kunden wies einen starken positiven Zusammenhang mit der persönlichen Leistungsfähigkeit, der Arbeitszufriedenheit sowie einen negativen Zusammenhang mit der Depersonalisierung der Beschäftigten auf
Rosenbaum (2009)	Restaurants	Nein	Qualitative Studie	*n* = 84 Kunden *n* = 2 Eigentümer *n* = 3 Management *n* = 27 Beschäftigte	Untersuchen die wechselseitige Unterstützung zwischen Dienstleistenden und ihren Kunden	*Beschäftigten erhalten von Kunden:* Emotionale Unterstützung, Freundschaft, erhöhte Trinkgelder, Loyalität, Geschenke *Kunden erhalten von Beschäftigten:* Individuelle Aufmerksamkeit, emotionale Unterstützung, Freundschaft, instrumentelle Unterstützung, Geschenke
Rosenbaum und Massiah (2007)	Fitnessstudios	Nein	Qualitative Studie und Quantitative Studie	*n* = 207	**UV: **Social-Emotional Support, Instrumental Support (von anderen Kunden) **AV: **Customer citizenship (Participation, Cooperation, Loyalty), Customer Care (Empathy, Responsibility)	Sowohl die sozial-emotionale Unterstützung als auch die instrumentelle Unterstützung von anderen Kunden erwies sich als signifikanter Prädiktor für das partizipative Verhalten von Kunden. Während sich der Zusammenhang zwischen sozial-emotionaler Unterstützung und der empathischen Anteilnahme gegenüber anderen Mitgliedern als signifikant erwies, konnte kein Zusammenhang zwischen der instrumentellen Unterstützung und der empathischen Anteilnahme gefunden werden
Shannahan et al. (2017)	Verschiedene business-to-business Unternehmen	Ja	Explorative und konfirmatorische Faktorenanalyse	*n* = 628	**UV**: customer organizational citizenship behaviour **AV**1: customer-involved sales performance **AV**2: salesperson behavioural performance **AV3: **salesperson outcome productivity	Die Ergebnisse zeigen u. a., dass das customer organizational citizenship behaviour (COCB) in einem positiven Zusammenhang mit der customer-involved sales performance (CISP) steht. Wenn Verkäufer das freiwillige Verhalten von Kunden wahrnehmen, kann sich dies positiv auf die Verkaufsleistung der Beschäftigten auswirken
Yi et al. (2011)	Elektronik Fachgeschäfte	Nein	Multilevel-Analyse	*n* = 332 Kunden *n* = 142 Dienstleistende *n* = 31 Manager	**UV**: Customer Participation behaviour, Customer Citizenship behaviour **AV**: Employee performance, Employee satisfaction, Employee Commitment, Turnover intention	Die Ergebnisse zeigen, dass das partizipative Verhalten der Kunden positive Auswirkungen auf die Leistung und das Engagement der Beschäftigten hat. Diese sind wiederum negativ mit der Kündigungsabsicht der Dienstleistenden assoziiert
Zimmermann et al. (2011)	Autohäuser	Nein	Längschnittsanalyse, Multilevel-Analyse	*n* = 421 Kunden *n* = 82 Dienstleistende	**UV/AV:** Positive Affect Customer, Customer-initiated Support, Positive Affect Employee, Positive Affect Customer	Die Ergebnisse der Studie zeigen, dass das unterstützende Verhalten von Kunden mit der positiven Stimmung von Dienstleistenden verbunden war. Die positive Stimmung des Mitarbeiters hatte wiederum einen positiven Einfluss auf die Stimmung des Kunden (vermittelt über das positive Service-Verhalten des Mitarbeiters)
Bednarek (2014)	Verschiedene „business-to-consumer“ Industrien: Einzelhandel, Gastronomie, Finanzservice, Friseur	Nein	Quantitative Befragung, Multilevel-Analyse	*n* = 141 Dienstleistende *n* = 375 Kunden	**UV: **customer demands (negative customer behaviours) and customer resources (positive customer behaviours) **AV:** frontline employees’ customer-oriented attitudes and customer-oriented behaviours	Die Ergebnisse belegen, dass die Auswirkungen negativer Verhaltensweisen von Kunden (z. B. verbale Aggressionen) auf die emotionale Erschöpfung von Dienstleistenden durch das positive Kundenverhalten (z. B. emotionale Unterstützung) moderiert wird. Damit nimmt nicht nur die soziale Unterstützung von Kollegen und Führungskräften, sondern auch die Unterstützung von Kunden eine entscheidene Rolle in der Prävention arbeitsbedingter Belastungen ein

**Table 6:**

Kategorie	Subkategorie	Zitat
1. Negative personenbezogene Kundenverhaltensweisen	1. Unfreundliche Kunden	„Was mich belastet mit den Gästen. Also es fängt an mit der Unfreundlichkeit auf jeden Fall. Das ist so eine Sache, die jedem Mitarbeiter hier schlechte Laune bringen kann, echt. Also wenn Gäste reinkommen, die sehr unfreundlich sind, die sehr frech sind“ (Beschäftigte Baumarkt)
	2. Respektlose Kunden	„Mir hat mal ein Auftraggeber gesagt: ‚Wissen Sie, Herr XY, Trainer sind für uns die Sklaven der Neuzeit. (…) Denen sage ich, was sie tun haben, und die tun, was ich ihnen sage‘“ (Beschäftigter Unternehmensberatung)
	3. Ungerechte & unfaire Kunden	„Wenn Leute betrügen oder eine Show abziehen oder arrogant sind, überheblich sind, unaufrichtig sind, schleimen, also sich wirklich einschleimen oder so was, also dann ist ganz aus, ganz vorbei“ (Beschäftigte Altenheim)
	4. Verbale Aggression	„Ich wurde bezeichnet als Hurensohn. Und dann wurde ich angespuckt mit so einem Ding als Mitarbeiter“ (Beschäftigter Fast-Food Restaurant)
	5. Sexuelle Aggression	„Ich hatte auch schon eine Kollegin, die auch so angebaggert wurde, die saß dann hinterher eine Stunde heulend im Pausenraum“ (Beschäftigte Baumarkt)
	6. Physische Aggression	„Die ERSTE Situation war mit einem Kunden, der einen Latte Macchiato bestellt hatte, der dann viel zu kalt war. Der hat dann den Mitarbeiter rausgerufen und wollte sich dann mit dem schlagen. […] Und die andere Interaktion war, wir hatten hier drinnen mal jemanden, der das ganze Tablett einfach […] auf mich geschmissen hat“ (Beschäftigter Fast-Food Restaurant)
2. Negative arbeitsbezogene Verhaltensweisen	1. Überzogene Kundenerwartungen	„Die Ansprüche der Kunden (…) haben sich verändert. Also unheimlich viele Sonderwünsche, ne? Also es wird ein Essen angeboten und dann sagt der Gast: ‚Ich hätte gerne kein Risotto, sondern dö-dö-dö-dö‘? Dann tauchen acht Sonderwünsche auf“ (Geschäftsführer Restaurant)
	2. (Illegitime) Kundenbeschwerden	„Du hast mir das das Medikament nicht gebracht.“ Und ich weiß hundertprozentig, dass ich es ihr gebracht habe. Und sie sagt denn eben: „Nein, das IST nicht so. Und ich werde mich beschweren“ (Beschäftigte Pflege)
	3. Mangelnde Wertschätzung	„Ja, das ist ein schwieriges Thema, weil die Wertschätzung vom Pflegekunden ist sehr selten. (…) Das erlebt man tatsächlich ein bisschen zu wenig, weil man oft tatsächlich viel tut für die Menschen. Und also da kommt wenig zurück. […] So explizite Wertschätzung ist extrem selten“ (Beschäftigte ambulante Pflege)
	4. Mangelnde Partizipation des Kunden	„Jemand, der schon in der Arbeitsvermittlung im Prinzip sich gar nicht meldet beziehungsweise zu Terminen nicht kommt oder nicht ans Telefon geht und keine Kooperation zeigt, den werde ich als Fallmanager auch nicht vermitteln können. Also es muss eine gewisse Bereitschaft sein zum Kooperieren und eben auch so ein Wunsch da sein: ‚Okay, es soll sich etwas in meinem Leben ändern‘“ (Beschäftigte Fallmanagement)
	5. Abstimmungsprobleme mit Kunden	„Ich hatte jetzt gerade einen dabei, der hatte Zement bestellt, wollte aber eigentlich Estrich haben. Sind zwei verschiedene Paar Schuhe. Da sage ich: ‚Ja, aber wenn du mir sagst, du willst Zement haben, dann schicke ich dir Zement und keinen Estrich. Dann musst du mir schon sagen, was du möchtest. (…) Also dann KOMMUNIZIERE mit mir, ja? Dann weiß ich auch, was du willst‘“ (Beschäftigte Baumarkt)
3. Traumatische Erfahrungen	1. Konfrontation mit Schicksal	„Ein sehr HEFTIGES Erlebnis, was ich jetzt in meiner langjährigen Berufslaufbahn hatte. Ich habe vor drei Wochen mit einer Kundin gemeinsam am Telefon geweint, weil sie mir sehr ans Herz gewachsen ist […] und sie mir erzählt hat, dass der Partner verstorben ist. Und da konnte ich dann nicht mehr“ (Beschäftigte Fallmanagement)
	2. Konfrontation mit Tod	„Ja, puh. Also ich sage mal so, so der Suizid, da bin ich nach Hause gekommen. Ich war leichenblass und ich habe das erste Mal einen toten Menschen gesehen und dachte: ‚Wow.‘ Und auch die Geschichte, die dahinterstand, hat einen traurig gemacht. Also ich habe mir dann den Abschiedsbrief durchgelesen. Und da war einem schon also die Tränen nah. Und, ja, das war schon so ein prägendes Ereignis. Also da erinnere ich mich jetzt nach sieben Jahren noch dran“ (Beschäftige Polizei)
	3. Konfrontation mit Krankheiten	„Und psychisch, ja, schon belastend. Wir haben viel mit extremen Situationen von Menschen zu tun. Und da ist es immer so bisschen die Schwierigkeit, die Gratwanderung zwischen Einfühlung und professioneller Distanz hinzubekommen? Also da habe ich schon verschiedenste Kollegen auch erlebt, die dann GAR nicht mehr die Arbeit machen konnten. Was wir halt immer wieder auch mal haben, wo Leute wirklich dann auch lebensgefährlich erkrankt sind. Wir haben ja auch Drogenkranke und schwere Alkoholiker (…) Das sind schon Dinge, die einem nahegehen“ (Beschäftigte Fallmanagement)
	4. Konfrontation mit Unfallereignissen	„Also ein Kollege, der hat mal, ja, ein schwerer Verkehrsunfall mit zwei toten Säuglingen. Und die Kollegen konnten dann auch nicht mehr, ne? Die haben dann auch Hilfe gebraucht, weil die sind auch Mutter und Vater gewesen. Und das reißt dann auch einfach MIT, ne?“ (Beschäftigter Polizei)
	5. Überbringung schlechter Nachrichten	„Also wenn dann wirklich ein Angehöriger dann irgendwie zusammenbricht, meinetwegen Kreislaufkollaps kriegt oder so was, dann ist das schon relativ belastend, ne? Also die letzte Nachricht, die ich überbracht habe, das war auch ein Junge, Anfang zwanzig, der hat sich vor den Zug gestellt und DIE [Mutter] ist uns da echt zusammengebrochen. Also das habe ich noch nicht erlebt. Die hat ganz, ganz laut geschrien“ (Beschäftigter Polizei)
4. Stressoren aus der Emotionsarbeit	1. Darstellungsregeln	„[Führungskraft:] Aber du weißt schon, ne, also so geht es jetzt aber nicht. Das musst du dem Kunden schon freundlich sagen.“ Und dann stehst du da, würdest am liebsten anfangen zu heulen, und sagst: „Ja, ich mache das.“ Aber ich kann das nicht. Bei den ersten hundert, oder hundertfünfzig Kunden, okay. Aber irgendwann kannst du nicht mehr das mit einem Lächeln im Gesicht sagen: „Lieber Kunde, wären Sie so lieb, würden den Wagen hier stehen lassen und hinter die Linie treten“ (Beschäftigte Baumarkt)
	2. Emotionale Dissonanz	„Na ja, ich unterdrücke meine Gefühle so lange, bis ich gegen die Wand schlage. […] Wenn ich zum Beispiel einen sehr nervigen Kunden habe, der dann halt mir auf die Pelle rückt“ (Beschäftigter Fast-Food Restaurant)
	3. Surface Acting	„Wir sind auf dem Zahnfleisch hier raus und auf dem Zahnfleisch wieder hier rein, und das über Wochen hinweg. Du kommst in den Markt rein, ziehst deine Klamotten an und spätestens an der Stempeluhr gehst du dann, DRRRING, das Lächeln ist im Gesicht. Und du kannst loslegen. Also in deiner Pause fällt dir wieder alles aus dem Gesicht, weil du einfach mal zwei Minuten abschalten kannst“ (Beschäftigte Baumarkt)
5. Unplanbarkeit von IA	1. Unwägbarkeit „Planbarkeit“	„Also du kannst jetzt nicht morgens sagen: ‚Ich mache das und das und das und DAS. Und dann bin ich fertig.‘ Das funktioniert nicht. Sondern da kommen eben halt noch dreißigtausend andere Sachen zwischendurch rein, die dich dann von deiner eigentlichen Arbeit wieder wegholen“ (Beschäftigter Betriebsgastronomie)
	2. Unwägbarkeit „Kundenhandeln“	„Also einmal haben wir den MENSCHEN als unkalkulierbares Gegenüber, dem WIR ja Dienst leisten SOLLEN“. (Experte Gastronomie)
6. Hohe Interaktionsintensität	1. Qualitative Anforderung IA	„Aber in dem Moment, wenn Sie merken, whoa, es funktioniert irgendwie nicht, dann müssen Sie sich VOLL einlassen, ja, das ist die anstrengendste Arbeit, weil Sie die Energie des anderen ja total aufnehmen und überlegen müssen, ey, ja, hm, das geht hier jetzt gerade nicht weiter, ne? Und wieso, weshalb? Das ist anstrengend für mich“ (Beschäftigter Unternehmensberatung)
	2. Quantitative Anforderung IA	„Im Drive-In finden sich dagegen sehr kurze Interaktionen mit einer hohen Taktung. Hierbei sind genaue Servicezeiten zu erfüllen, innerhalb derer Gäste bedient werden müssen. Die Beschäftigten erleben die Servicezeiten als unrealistisch und stressig.“ (Beobachtungsprotokoll Fast-Food Restaurant)

**Table 7:**

Kategorie	Subkategorie	Zitat
1. Positive arbeitsbezogene Verhaltensweisen	1. Partizipatives Verhalten	„Es gab schon mal einen Fall, da hatten wir einen Gast, der wollte nur was zum Mitnehmen, aber der eine Bestellung an den Tisch gebracht, weil mein Kollege gerade keine Zeit hatte“ (Beschäftigter Fast-Food Restaurant)^a^
	2. Unterstützung durch Informationen	„Du hast Kunden, die kommen und sind vorbereitet, ne? Wahnsinn. Die haben schon alle Artikelnummern zu Produkten rausgeschrieben … wo du dann denkst: ‚Mein Gott, das ist so cool, wenn jeder Kunde so ein Stück davon hätte, wäre allen schon geholfen‘“ (Beschäftigte Baumarkt)
	3. Unterstützung durch Feedback	„Ich freue mich über jedes positive Feedback, was ich bekomme, und versuche, das dann auch für mich mitzunehmen. Und natürlich auch ehrliches Feedback vom Kunden. Wenn er sagt: ‚Das und das hätte besser laufen können‘, versuche ich das jetzt nicht unbedingt als Meckern aufzufassen, sondern: ‚Okay, wir können an unserem Service vielleicht noch schrauben. Wir können noch mehr Kundenzufriedenheit herstellen.‘ Aber wenn ich natürlich positives Feedback bekomme, fühle ich mich wie ein König, ne?“ (Beschäftigter Betriebsgastronomie)
	4. Wertschätzung der Arbeit durch den Kunden	„Bei den Flüchtlingen ist es ganz anders, und das finde ich sehr, sehr SCHÖN, die Leute haben immer ein Lächeln auf den Lippen. Die sagen tausendmal Dankeschön, auch wenn sie sich eigentlich gar nicht bedanken müssten, ja? Also WIR spüren IMMER die Wertschätzung der Flüchtlinge uns gegenüber“ (Beschäftigter Fallmanagement)
	5. Verständnisvolle Kunden	„Aber es gibt auch Gäste, die haben Verständnis bei stressigen Situationen und sagen zum Beispiel: ‚Alles gut, ne? Sie sind auch nur Menschen, ne?‘ Stell dir vor, vor vier Tagen sind mir zwei Getränke runtergefallen in der Lobby, ne? War eine scheußliche Situation. Und auch der Gast hat einfach gemeint: ‚Kann passieren. Ist alles gut, ne?‘“ (Beschäftigter Fast-Food Restaurant)
2. Positive personenbezogene Verhaltensweisen	1. Nette & freundliche Kunden	„Ich finde, was bei mir IMMER hängenbleiben wird, ist die Freundlichkeit der Gäste. Es gibt so richtig nette Gäste, weißt du? Da drauf kannst du nicht anders reagieren, außer SEHR viel zu lächeln“ (Beschäftigter Gastronomie)
	2. Freundschaftliches Verhältnis	„Und der Witz ist ja, dass viele dieser Menschen ja BLEIBEN. Die melden sich immer wieder. Das heißt, es gibt Leute, die kenne ich jetzt seit zwanzig Jahren. (…) Und manche sind zu Freunden geworden, auch zu Freunden der Familie“ (Beschäftigter Unternehmensberatung)
	3. Emotionale Unterstützung von Kunden	„Und manchmal sehen die das auch, wenn eine Pflegekraft auch mal schlecht gestellt ist. Da wird auch mal nachgefragt. Wenn man dann sagt: ‚Mensch, nein, heute ist mir echt nicht so gut … ach, ich habe so viel im Kopf‘. Dafür bringen die dann auch ein bisschen Verständnis auf. Da versuchen sie einen abzulenken oder sagen dann auch mal: ‚Ach, weißt du, ICH habe auch schon mal Liebeskummer gehabt, ne‘“ (Beschäftigte ambulanten Pflege)
3. Ressourcen aus der interaktiven Tätigkeit	1. Grund Berufswahl „Mit Menschen arbeiten“	„Und habe das Ganze ja irgendwann einmal hingeschmissen, eben aufgrund der Tatsache, weil ich festgestellt habe, ich will mit Menschen arbeiten. Und da bin ich jetzt auch gelandet, Gott sei Dank. Ja, und von daher ist es tatsächlich ein Traumjob. Ne, wo ich sage, okay, das ist meine Berufung, Und deswegen macht der mir unglaublich viel Freude und kann mir auch nichts anderes mehr vorstellen“ (Beschäftigter Unternehmensberatung)
	2. Spaß bei der Arbeit mit Kunden	„Deren Lächeln, deren Zufriedenheit, sagen wir mal so. Ich bin glücklich, wenn mein Kunde glücklich ist. Wenn ich sehe, der Kunde lacht, der Kunde redet höflich mit mir, er lacht mit mir, er hört auf meine Empfehlungen, das macht mich auch sehr glücklich über meine Kunden.“ (Beschäftigter Baumarkt)
	3. Aus IA etwas selbst mitnehmen	„Ja, man lernt von den Bewohnern […] so Lebensweisheiten manchmal. Dass man immer mit dem glücklich sein soll, was man teilweise hat. Und die zeigen ja auch, dass man echt es schlechter haben kann. Also doch so Lebensweisheiten und so Sprüche, doch“ (Beschäftigte Altenheim)
	4. Sinnerleben bei der Arbeit	„Meine Arbeit ist per se sinnvoll. Sonst würde ich sie nicht tun. Also der Sinn, der zeigt sich in jeder SEKUNDE eigentlich, ja, warum ich das mache“ (Beschäftigter Unternehmensberatung)
	5. Abwechslungsreiche Arbeit	„Es hat mir enorm viel Spaß gemacht. Es ist ja sehr vielfältig und man arbeitet mit SO vielen Menschen pro Tag und mit verschiedenen Menschen pro Tag“ (Beschäftigter Gastronomie)

^a^Die Satzstruktur der Zitate wurde teilweise der Verständlichkeit halber leicht geändert. An der inhaltlichen Aussage wurden keine Veränderungen vorgenommen

**Table 8:**

Kategorie	Subkategorie	Zitat
1. Gestaltungsfaktor Arbeitsaufgabe	1. Ausreichender Interaktionsspielraum	I: „Also Ihr Gestaltungsspielraum ist Ihnen schon sehr wichtig?“ B: „Ja, dass ich einfach meine Gesprächsführung so gestalten kann, wie ich auch merke, dass es gut für mich und für den Prozess der Beratung funktioniert“ (Beschäftigter Fallmanagement)
	2. Reduzierung widersprüchlicher Anforderungen	„Ja, wie gehen wir mit Unterbrechungen um? Das ist in dem letzten Jahr schon extrem gewesen. Der Verkäufer ist mit einem Kunden in einem Fachgespräch drin, die anderen Kunden haben wenig Zeit und machen natürlich Druck im Hintergrund. Deswegen schaffen wir jetzt Beratungskojen. Das heißt also, wenn der Kunde eine Badsanierung haben möchte, zieht sich der Verkäufer mit dem Kunden zurück“ (Geschäftsführer Baumarkt)
	3. Interaktionsgerechte Qualifizierung	„Wir machen viele Rollenspiele. Also auch Umgang mit Sterbenden, mit Patienten, mit Angehörigen. Das machen wir schon alles. Wir überlegen uns so ein paar Kommunikationstaktiken“ (Beschäftigte Altenheim)
	4. Variabilität von interaktiven und monologischen Tätigkeiten	„Was ich auch gut finde, ist diese Mischung. Also ich würde sicher nicht hier den ganzen Tag acht Stunden lang Beratungsgespräche machen können. Also das würde mich kaputtmachen, glaube ich, tatsächlich. Ich habe auch gemerkt, dass ich nicht mehr als VIER Menschen am Tag beraten kann. Das war einfach so eine Grenze. Diese Aufteilung von Verwaltungsarbeit und Beratung, diese Mischung, die ungefähr 50/50 ist, die macht es mir leicht, das relativ lang gut machen zu können, ohne dass es mir schlecht geht“ (Beschäftigte Fallmanagement)
2. Gestaltungsfaktor Arbeitsorganisation	1. Ausreichende Zeit- und Personalbemessung	„Das Menschliche kommt einfach gerade zu kurz. Das ist natürlich auch eine gewisse Art, dass man sich gegenseitig etwas gibt. Dass sich die Patienten nicht wie Marionetten fühlen. Dass man die Patienten nicht wie am Fließband abfertigt. Durch den Personalmangel ist das natürlich die größte Belastung. Durch den Personalmangel wird man seinen eigenen Ansprüchen nicht gerecht, den Patienten nicht mehr gerecht. Man selber ist unzufrieden, weil man so nie selber arbeiten wollte“ (Beschäftigte Altenheim)
	2. Angemessene Gratifikation von Interaktionsarbeit	„Berufe, in denen Interaktionsarbeit geleistet wird, sind nach wie vor in der Wertschätzung am unteren ENDE. Während körperliche Arbeit früher schon immer bewertet wurde z. B. Schmutzzulage, ist Interaktionsarbeit nie eine Arbeit gewesen, die mit Geld bezahlt wurde. Und wenn jetzt klar ist, auch das ist Arbeit, das IST Arbeit und die hat ihren Wert und die muss auch bezahlt werden, dann würde das ja eventuell zwangläufig irgendwann dazu führen, dass die Jobs besser bezahlt werden“ (Expertin Einzelhandel)
	3. Interaktionsgerechte Pausengestaltung	„Im Kern brauche ich dann meine Ruhe. Also keine Interaktionsarbeit mehr. Weil es muss ja mal Pause sein. Und das heißt bei uns, keine Interaktion“ (Beschäftigter Unternehmensberatung)
	4. Kundenorientierte Gestaltung betrieblicher Prozesse und Strukturen	„Sie können ein Top-Produkt haben, wenn die Interaktion schlecht war, wird trotzdem schlecht geredet werden. Jetzt können Sie aber folgendes haben: Sie können ein schlechtes Produkt haben. Wenn Sie eine gute Interaktion aber haben, ein gutes Beschwerdemanagement im Nachgang, dann wird er nachher sagen: „Mensch“, er hat zwar erst ein schlechtes Produkt gekriegt, aber der Mitarbeiter war freundlich, hat sich sofort gekümmert, hat das Produkt ausgetauscht. Dann gehen Sie nachher raus und sagen: ‚Es war super. Und das heißt, Sie können halt über die Interaktion sogar ein schlechtes Produkterlebnis ausgleichen‘“ (Geschäftsfrüher Betriebsgastronomie)
3. Gestaltungsfaktor Arbeitsumgebung & Arbeitsmittel	1. Förderliche physische Umgebungsfaktoren	„Wir hatten die Möglichkeit, Sparziergänge mit dem Kunden zu machen und im Park dann über die Dinge zu sprechen, die die Kunden bewegen, um einfach aus diesem für manche Kunden, ja durchaus auch, ich sage jetzt mal, so angstbesessenen Raum von BEHÖRDE rauszukommen … dieses Verwaltungsgebäude … graue Flure … Behörde ist Behörde. Vielen Menschen macht das Angst. Ne? Und das kann durchaus eine Möglichkeit sein, dass sich Kunden eher öffnen“ (Geschäftsführerin Fallmanagement)
	2. Interaktionsdienliche Arbeitsmittel	„Die haben intuitiv dann, bevor sie losgelaufen sind, die App geöffnet, haben geguckt und haben eingegeben: ‚Mutternsprenger.‘ JA, 8,50 €. […] Die meisten Fragen sind ja: ‚HABEN Sie das? Wo ist das? Was KOSTET das?‘ Und damit praktisch 80 bis 90 % der Kundenfragen beantworten.“ (Geschäftsführer Baumarkt)
4. Gestaltungsfaktor Führung, Kollegium & Kultur	1. Soziale Unterstützung durch KollegInnen	„Dieses danach sich mit Kollegen zusammensetzen und einfach nur mal über das Erlebte so am Tag zu sprechen, der eine erzählt denn: ‚Mensch, ich hatte heute so einen Fall‘, und dann spricht man darüber und man spricht mit den Kollegen darüber. Das hat immer ganz, ganz viel gebracht, als wenn man das alles so ohne Gespräche mit nach Hause nehmen müsste“ (Beschäftigter Polizei)
	2. Interaktionsorientierte Führung	„Aber ich hatte auch schon eine Marktleitung bei mir dann, der dann den Herrn freundlich gebeten hat, das Haus zu verlassen und nie wiederzukommen, wo ich dann auch gesagt habe, das ist das, was ich mir wünsche. Dass ich wirklich komplett Rückendeckung kriege, dass sich meine Assistenz vor mich stellt und sagt: ‚Du, pass auf, bis hierhin und nicht weiter‘“ (Beschäftigte Baumarkt)
	3. Förderliche Organisationskultur für Interaktionsarbeit	„Wir haben auch ein Leitmotiv, was eben heißt, wir wollen mit zufriedenen, gut ausgebildeten Mitarbeitern das bestmögliche Restauranterlebnis erzielen, ja? Deswegen sage ich, bei uns steht der Mitarbeiter an erster Stelle und dann kommt erst der Gast. Weil ich kann natürlich nachher ständig meinem Mitarbeiter erzählen, der Kunde oder der Gast ist König, wenn ich meine Mitarbeiter dabei nicht gut behandle, werden sie auch den Gast nicht gut behandeln. Je besser der Mitarbeiter ausgebildet ist, je zufriedener er ist, umso besser ist der Service!“ (Geschäftsführer Fast-Food Restaurant)

## Footnotes

1 Die Bezeichnung der KundInnen schließt ebenfalls weitere betriebsexterne Personengruppen wie PatientInnen, KlientInnen und Lernende mit ein.

2 Die durchgeführte Studie fand im Rahmen des BMBF geförderten Drittmittelprojekts „Interaktionsarbeit: Wirkungen und Gestaltung des technologischen Wandels“ – (InWiGe) statt. InWiGe verfolgt als wissenschaftliches Begleitprojekt des Förderschwerpunkts „Zukunft der Arbeit: Arbeiten an und mit Menschen“ die Zielstellung, einen analytischen, konzeptionellen und empirischen Beitrag zur menschengerechten Arbeitsgestaltung von Interaktionsarbeit zu leisten.

3 Da sich das Forschungsfeld als recht heterogen darstellte und spezifische Stressoren und Ressourcen größtenteils nicht in Zusammenhang mit dem Begriff der Interaktionsarbeit gebracht wurden, erwies sich das Vorgehen einer rein systematischen Literaturanalyse als wenig zielführend. Um interaktionsbezogene Stressoren und Ressourcen zu identifizieren, die nicht explizit mit dem Forschungsgegenstand der Interaktionsarbeit in Zusammenhang gebracht wurden, wurde zusätzlich auf das methodische Vorgehen des Schneeballsystems zurückgegriffen.

4 Die Auswahl der Beschäftigtengruppen erfolgte im Rahmen des Projekts InWiGe (Quelle: www.interaktionsarbeit.de).
